# Robust isolation protocol for mouse leukocytes from blood and liver resident cells for immunology research

**DOI:** 10.1371/journal.pone.0304063

**Published:** 2024-08-22

**Authors:** Dorien De Pooter, Ben De Clerck, Koen Dockx, Domenica De Santis, Sarah Sauviller, Pascale Dehertogh, Matthias Beyens, Isabelle Bergiers, Isabel Nájera, Ellen Van Gulck, Nádia Conceição-Neto, Wim Pierson

**Affiliations:** 1 Infectious Diseases Discovery, Infectious Diseases Therapeutic Area, Janssen Research and Development, Beerse, Belgium; 2 Charles River Laboratories, Beerse, Belgium; 3 Discovery Technologies & Molecular Pharmacology, Therapeutics Discovery, Janssen Research and Development, Beerse, Belgium; 4 Infectious Diseases Discovery, Infectious Diseases Therapeutic Area, Janssen Research and Development, California, Brisbane, United States of America; Centers for Disease Control and Prevention, UNITED STATES OF AMERICA

## Abstract

Research on liver-related conditions requires a robust and efficient method to purify viable hepatocytes, lymphocytes and all other liver resident cells, such as Kupffer or liver sinusoidal endothelial cells. Here we describe a novel purification method using liver enzymatic digestion, followed by a downstream optimized purification. Using this enzymatic digestion protocol, the resident liver cells as well as viable hepatocytes could be captured, compared to the classical mechanical liver disruption method. Moreover, single-cell RNA-sequencing demonstrated higher quality lymphocyte data in downstream analyses after the liver enzymatic digestion, allowing for studying of immunological responses or changes. In order to also understand the peripheral immune landscape, a protocol for lymphocyte purification from mouse systemic whole blood was optimized, allowing for efficient removal of red blood cells. The combination of microbeads and mRNA blockers allowed for a clean blood sample, enabling robust single-cell RNA-sequencing data. These two protocols for blood and liver provide important new methodologies for liver-related studies such as NASH, hepatitis virus infections or cancer research but also for immunology where high-quality cells are indispensable for further downstream assays.

## Introduction

The liver is an important gatekeeper for many metabolic, hematologic and immunological processes, namely: enriching the bloodstream with nutrients absorbed by the gut and secreting proteins, such as complement and albumin which are vital for health, while also depleting and inactivating waste products and toxins from the circulation [1]. Immune homeostasis is maintained in the liver, not by excluding immune cells, but by generating an environment that dampens triggers that may otherwise put them on high alert [1]. Specialized cell types, such as Kupffer cells, liver sinusoidal endothelial cells (LSECs) and stellate cells are found in the intra-hepatic environment, alongside the more common T, B, NK and dendritic cells (DC) [2]. To extract immune cells from various tissues of interest, immunologists traditionally use a combination of a syringe plunger and cell strainers of various pore sizes. Although capturing lymphocyte singlets that roam the body freely is trivial, extracting unscathed cells from tumors and solid organs such as the liver requires a gentler approach, often requiring a combination of both mechanical and enzymatic treatments [3,4]. This latter approach has been shown to be required for capturing larger and more morphologically complex cell types, which are characterized by their large array of adhesion molecules and close interaction with the extracellular matrix [5,6]. Inadvertent damage, death, or the inability to even release these cells from their neighbors in the tissue environment impedes our ability to study them [5,6].

**Fig 1:**
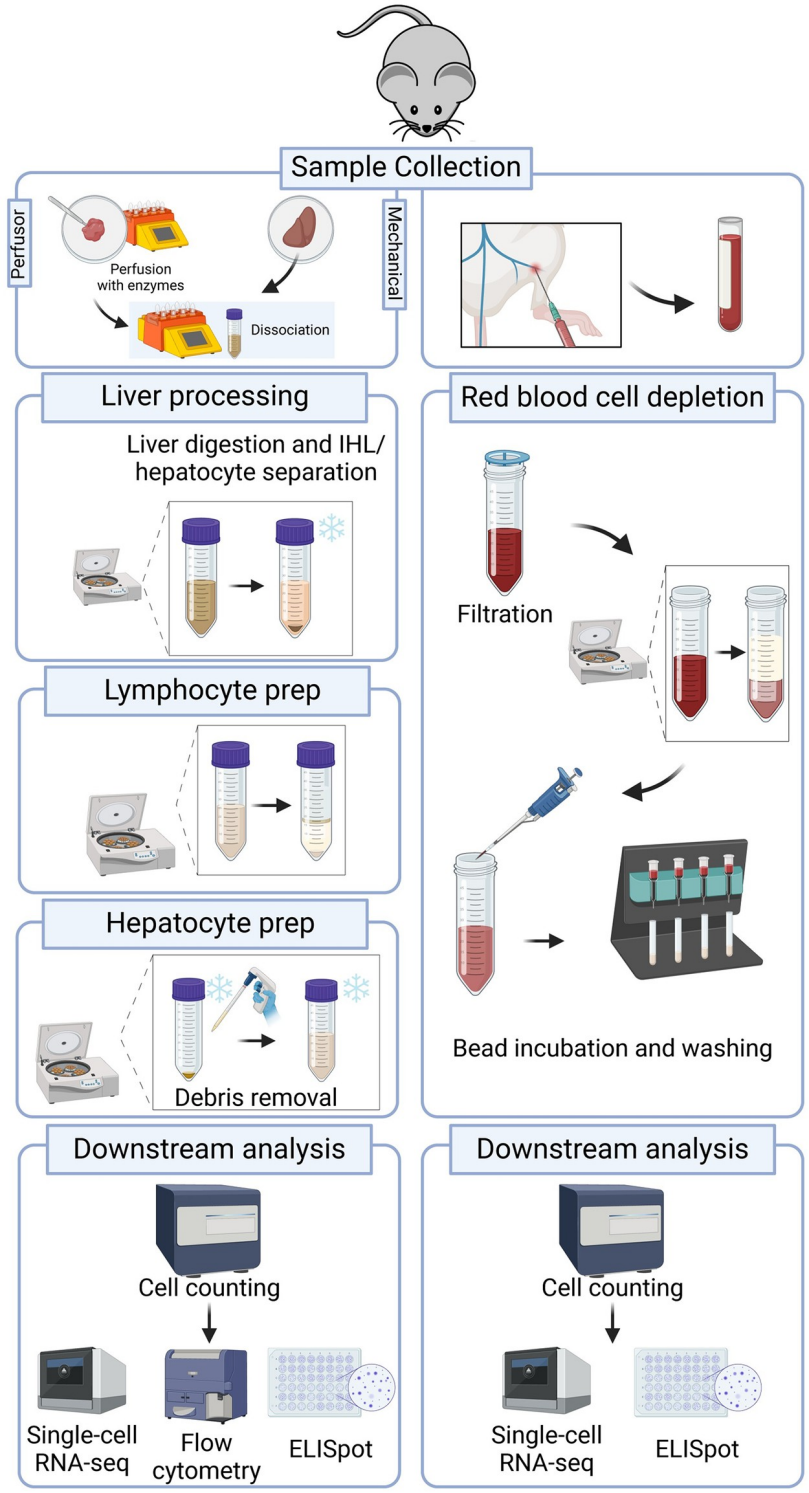
Protocol overview. After sample collection, both blood and liver are processed. Left lateral liver lobe is cut and digested on a GentleMACS Dissociator followed by centrifugation separation of hepatocytes and intra-hepatic cells. Lymphocytes are purified using a Percoll gradient to remove debris and remaining hepatocytes. Hepatocytes are pelleted for debris removal. After cell counting, cells are ready for all downstream applications. The blood samples are collected from the saphenous vein, after a filtering step, blood is incubated with magnetic beads for red blood cell depletion. After washing, cells are counted and ready for downstream applications. Image created with BioRender.

In the context of liver disease studies, we present a protocol framework that allows a parallel assessment of hepatocyte parameters and the functional state of the liver resident versus the peripheral immune cells (e.g., gene expression and protein expression) (Fig 1). Where blood sampling and PBMC isolation in clinical studies is a routine procedure, laboratory rodents have, due to their small size, only a restricted volume of blood available for both serological and cellular assays [7]. Given this limited sample, it is imperative that it is free of components that may obfuscate the biological processes at play, such as platelets and red blood cells, needlessly driving up the complexity and cost of downstream assays and analyses [8].

Using recently developed improvements in cell dissociation and isolation technologies, our approach aimed at striking a balance between sample integrity and throughput. Democratization of single-cell sequencing technologies now allows unprecedented insights into the workings of complex organs, revealing the transcriptional state of cells [9,10]. The use of single-cell RNA-sequencing has been especially valuable for detecting and understanding rare cell types, since their signal would easily be missed using bulk approaches [11]. In addition, this technique can also help understand complex immune responses at the population and individual cell level, which is a valuable tool for identifying novel targets for drug discovery. We herein describe optimized and robust, reproducible protocols for isolation of liver intra-hepatic immune and non-immune cells that can be successfully used for single-cell RNA-sequencing, flow cytometry and short-term primary mouse hepatocyte cultures, as well as for blood leukocytes, which are available in limited number in mouse samples [9,10].

We applied these protocols on naïve mice and on the liver-infection mouse model, an immune competent model transduced with an adeno-associated viral (AAV)-vector able to induce long term hepatitis B virus (HBV) viral persistence that mimics chronic human HBV infection [12].

We optimized a framework for isolation of cells both from blood and liver tissue, resulting in high quality cell suspensions that can be used as input for multiple downstream applications, which could positively impact the number of animals required for modelling liver diseases.

## Results

### Novel isolation protocol preserves liver immune cell composition and viability

**Fig 2:**
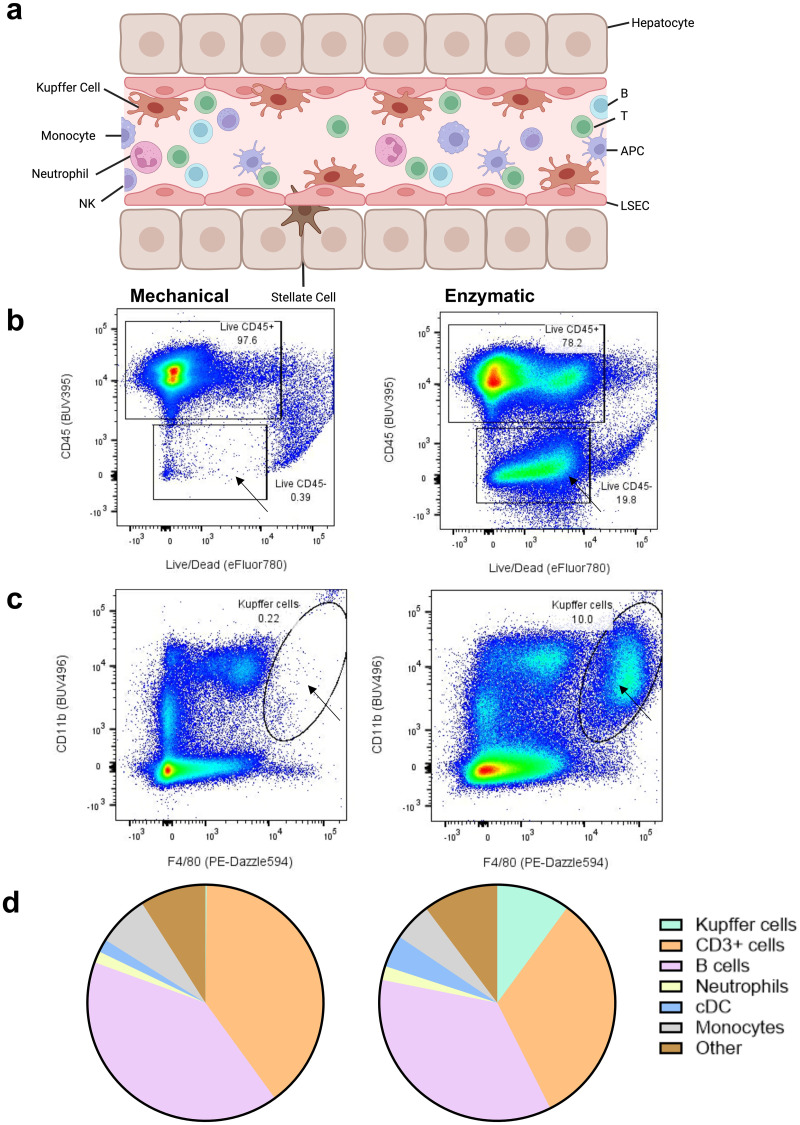
Flow cytometry to identify liver resident cell population composition after enzymatic digestion (right) compared to mechanical disruption (left). (a) Graphical representation of cell populations theoretically present in mouse liver (Image created with BioRender) (b) Dot plot for living (APC-Cy7) CD45+ (BUV395) cells shows an extra population of CD45 negative cells (indicated with an arrow). (c) Dot plot showing the Kupffer cell population (indicated with arrow) which are identified as CD11b+ (BUV496) and F4/80+ (PE-Dazzle594) double positive cells of living CD45+ population. (d) Pie chart showing cell populations within the living CD45+ cells (n = 5).

Since liver parenchyma consists mainly of hepatocytes and they are often a key (impacted) target in liver diseases, it is paramount to purify them with excellent viability (Fig 2a) [13]. In order to obtain a reproducible way to isolate mouse resident liver immune cells, liver was either manually perfused with saline, followed by mechanical dissociation or by a more gentle, (at the time of these experiments) pre-commercially available liver purification method using GentleMACS perfusors that clamps down the tissue and delivers buffers via a peristatic pump. This opens the extracellular matrix by applying enzyme mixes that break down the matrix so that all cells can escape at a later stage when both the mechanical-only and the perfusor-treated liver samples are subjected to a mild mechanical disruption by means of a C-tube dissociator (Fig 1).

Cells were purified using a centrifugation step to separate the large hepatocytes from non-parenchymal cells (NPCs), taking advantage of their differing sedimentation speeds (Fig 1). Afterwards, both hepatocytes and NPCs were further purified using cell debris removal kit for the hepatocytes, and Percoll gradient for the NPCs to finally result in a collection of all intrahepatic lymphocytes (IHLs).

After purification of the NPCs, we assessed cell yield, viability, and color of the cell suspension (S1 Fig). In comparison, the average viability of non-hepatic cells obtained via mechanical isolation (80.1% ± 11.8 Standard Deviation (SD)) was lower compared to the perfusor method (93.4% ± 1.7 SD) and the number of viable cells exceeding 2 million only when using the latter method. As an immediate consequence, in order to obtain the needed number of cells for downstream studies (typically one million cells are used in flow cytometry assays), the mechanical method requires the full mouse liver as input, whereas the enzymatic method only needs (the equivalent of) one lobe. The color of the suspension was redder for the mechanical method (S1 Fig) indicating that red blood cells were present whilst these were removed or washed away during the enzymatic Miltenyi procedure.

To characterize the purified NPCs resulting from the two methods, multicolor flow cytometry was performed. A cell population, likely enriched in LSECs (based on size and lack of CD45 staining, no LSEC-specific marker such as CD31 was included in this panel), was observed in the enzymatic isolation samples (30.2% ± 10.8 SD), but not in the mechanical samples (0.34% ± 0.077 SD) (Fig 2b). When looking further into the living CD45+ cells, we could identify a population of Kupffer cells, defined as CD11b+ and F4/80+ (Fig 2c and 2d), which were absent in the mechanical digestion samples (10.1% ± 1.74 SD versus 0.19% ± 0.071 SD). Finally, 2.5 times more conventional DCs (cDC) were present after perfusor-mediated digestion than after mechanical method (Fig 2d).

**Fig 3:**
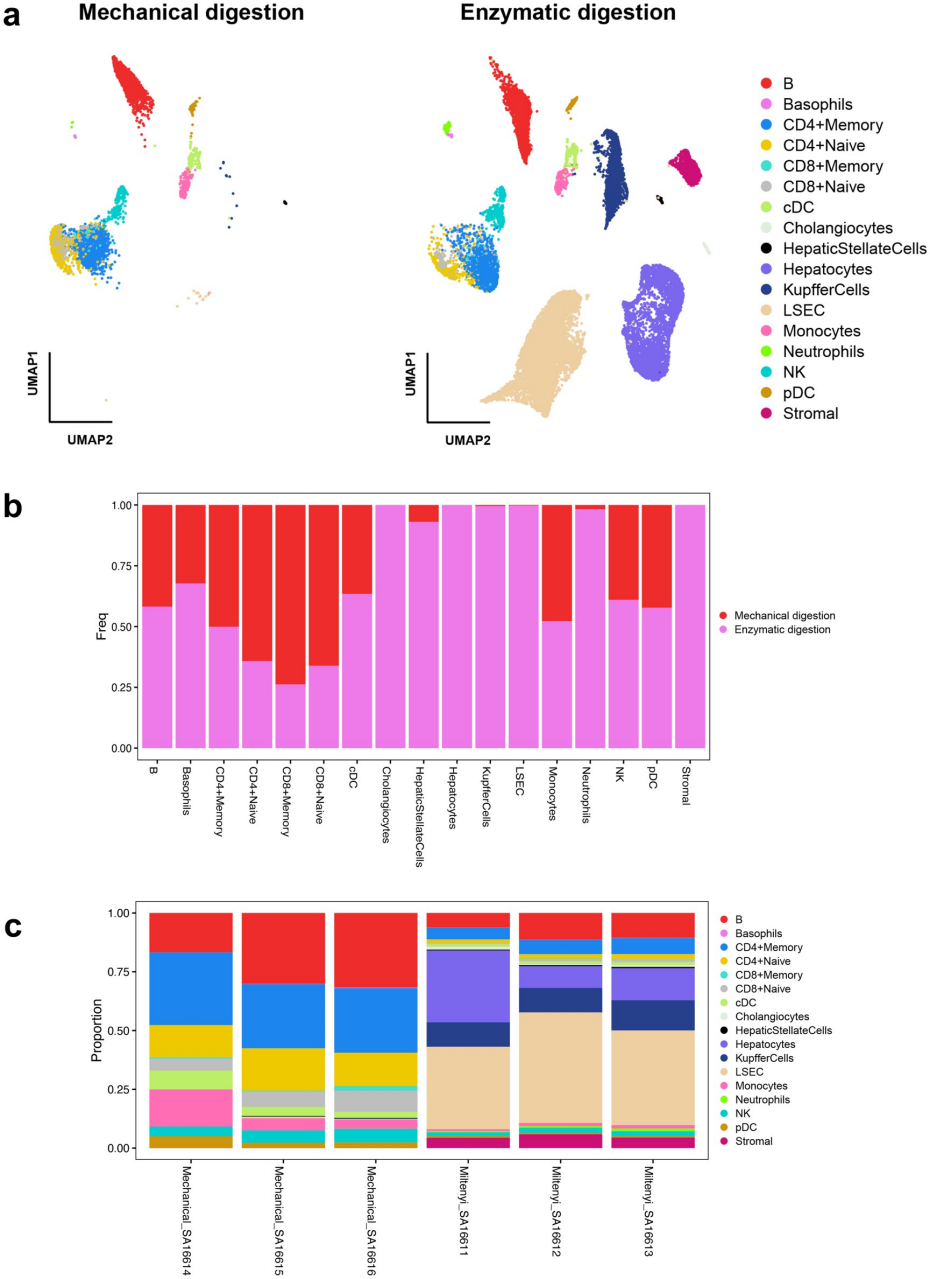
Single-cell RNA sequencing data for comparison of mechanical with enzymatic digestion of mouse liver. (a) UMAP projection of the cell population annotations across the two liver dissociation methods. (b) Bar plot with cell population proportions across the two liver dissociation methods. (c) Bar plot with cell population proportions across the different samples.

Moreover, the flow cytometry results were further confirmed using single-cell RNA-sequencing that besides Kupffer cells, other resident cells such as hepatocytes, cholangiocytes, LSECs and hepatic stellate cells can be efficiently detached from extracellular matrix via enzymatic digestion (Fig 3a–3c, S4 and S5 Figs).

In the immune cell compartment, both methods were able to detect B cells, basophils, CD4 and CD8 expressing T cells, cDC, monocytes, NK cells, pDCs, and neutrophils, although the last-mentioned were more abundantly detected (168 versus 3 cells) in the enzymatic digestion samples (Fig 3b, Table 1). Differences in proportions were observed across the evaluated methods, since the liver resident non-immune cells were well captured in the perfusor method whereas the mechanical dissociation captures predominantly T cells (Table 1). Downstream single-cell RNA-sequencing data on liver dissociates from both evaluated protocols, shows that the enzymatic digestion was able to capture more high-quality cells (S2 and S3 Figs).

**Table 1:** Cell numbers distribution of the different cell populations per liver isolation protocol. The aggregated sum per cell type for each protocol is shown An overview of the cell type annotations can be found in S5 Fig.

	Mechanical	Miltenyi
**B**	1332	1855
**Basophils**	10	21
**CD4+Memory**	1202	1197
**CD4+Naive**	667	372
**CD8+Memory**	62	22
**CD8+Naive**	352	180
**cDC**	140	243
**Cholangiocytes**	0	168
**HepaticStellateCells**	7	94
**Hepatocytes**	0	3924
**KupfferCells**	10	2286
**LSEC**	18	8260
**Monocytes**	207	226
**Neutrophils**	3	168
**NK**	240	375
**pDC**	109	149
**Stromal**	0	996
***Total***	***4359***	***20536***

### Functional intra-hepatic lymphocytes retain their immune recall capacity

To enable further studies to characterize the immune liver environment in different stages of disease or upon treatment we sought to understand the functionality of the cells after isolation. The lymphocytes that were enriched in the supernatant after the first centrifugation were collected and further purified to remove debris and residual hepatocytes (Fig 1). The upstream introduction of the enzymatic digestion protocol allowed for substantial improvements of the data quality of IFN-γ ELISpot used to study the immune recall after treatment with a therapeutic DNA vaccine in preclinical models as previously published [14]. Using the isolation protocol described in Fig 1, only 7 out of 137 samples were rejected based on non-specific responses to solvent-only conditions whereas previously 100 out of 321 samples needed to be rejected downstream of the mechanical dissociation.

### Enzymatic digestion improves hepatocyte viability and allows for ex vivo primary cell culture

**Fig 4:**
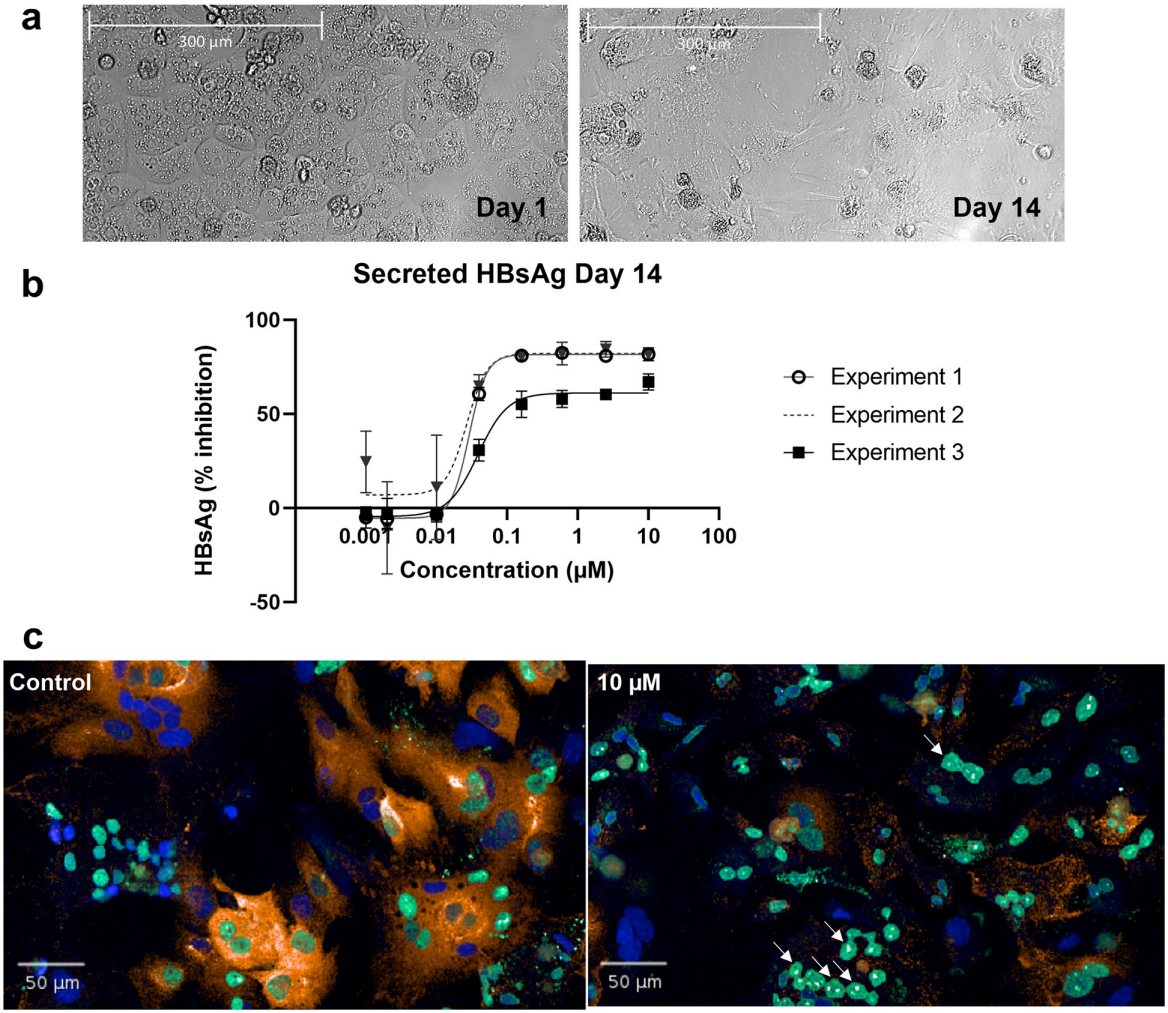
Primary mouse hepatocytes can be used for antiviral compound testing using confocal imaging and HBsAg CLIA. (a) Culture of primary mouse hepatocytes from AAV-HBV infected mice can be maintained for at least 14 days and (b) can be used for ex vivo compound screening of antiviral compounds which show an inhibition of HBsAg in the supernatant of the hepatocyte culture in three independent experiments and (c) induced speckling of HBc visualized by confocal imaging in which Hoechst is visualized in blue, HBsAg is colored in orange (CF568) and core protein is stained in green (AlexaFluor488).

To obtain hepatocytes with high viability from the liver cell suspension, the first step was a low-speed centrifugation in which the large and heavy hepatocytes sink disproportionally fast. These freshly harvested hepatocytes were then plated and kept in culture for up to two weeks (Fig 4a). We investigated whether hepatocytes from the AAV-HBV preclinical model of hepatitis B were conducive to this procedure, are viable and can be subsequently treated with antiviral compounds and used to assess the effect on the hepatitis B surface antigen (HBsAg) and e antigen (HBeAg) expression. To that end, three independent experiments of a 14-day assay with a capsid assembly modulator (CAM-A class) showed reproducible inhibition of secreted HBsAg in the supernatant of the hepatocyte culture, measured using a chemiluminescence immunoassay (CLIA) (Fig 4b), and a speckling pattern of Hepatitis B core antigen (HBc) was identified using confocal imaging (Fig 4c).

The freshly harvested hepatocytes were also cryopreserved in liquid nitrogen vapor for a short period of time and cellular performance after thawing was compared between 1 and 5 weeks of storage (S1 Table). Based on a cellular staining with trypan blue, recovery of viable cells that were stored for 1 week was ≥90% in two different freezing media conditions, while cells that were thawed after 5 weeks showed a drop in viability below 90%, in addition, morphology of the cells was perturbed as compared to freshly seeded cells, and inconsistent antiviral responses after a multiday assay follow-up were observed (S6 Fig). perfusor-aided digestion of mouse liver yields viable hepatocytes ready for cell banking and further downstream assays such as confocal imaging weeks after seeding.

### Red blood cell depletion leads to optimal leukocyte preparation from mouse whole blood

**Fig 5:**
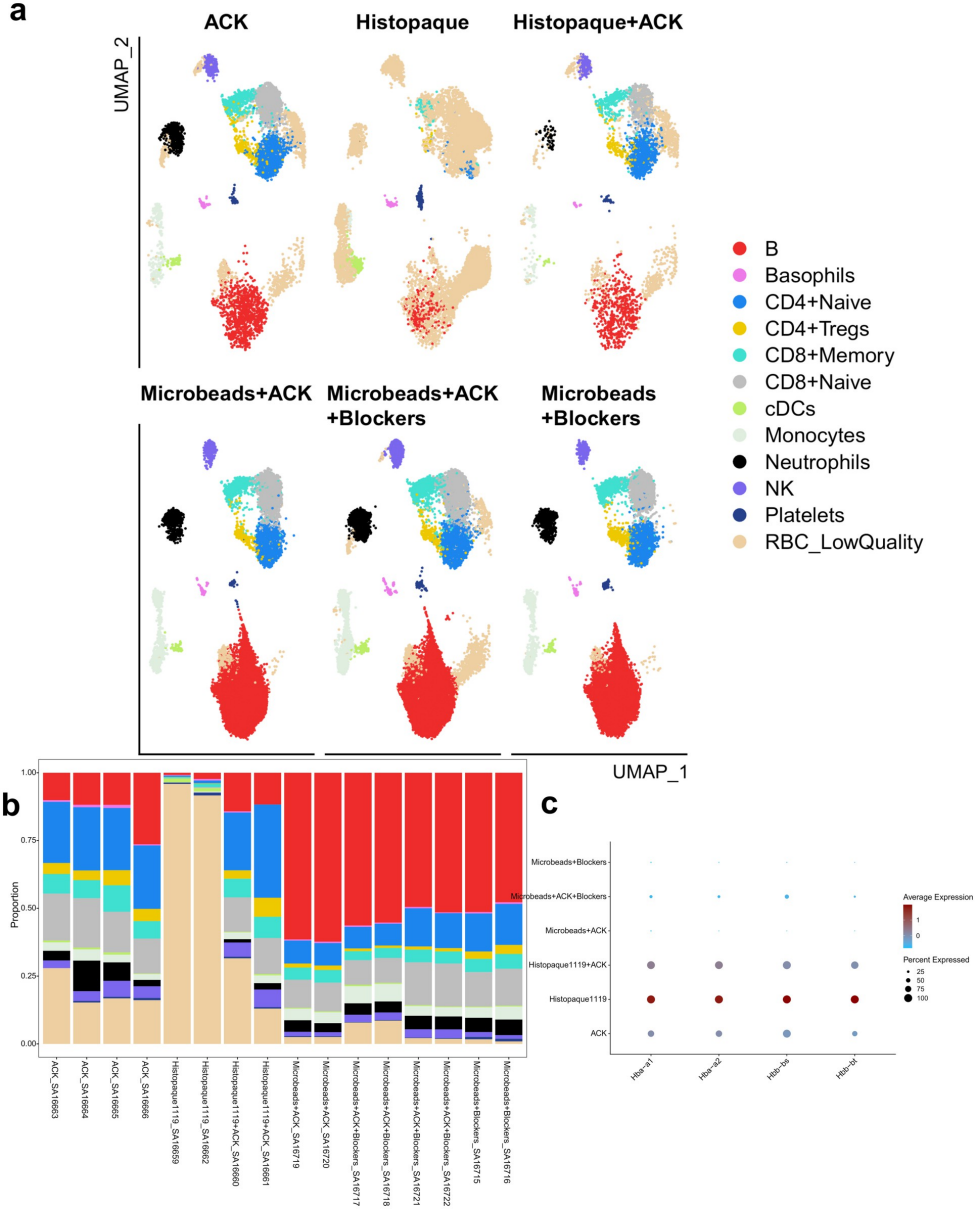
Single-cell sequencing data from comparison of protocols for purification of whole blood. (a) UMAP projection of cell types across the different whole blood purification methods (b) Bar plot with cell population proportions across the different whole blood purification methods. (c) Dot plot visualization of scaled expression of the contamination of the different red blood cell markers per protocol. The size of the dots represents the percentage of cells expressing the marker across all cells from the condition. An overview of the cell type annotations can be found in S9 Fig.

Prior to liver sampling, whole blood was collected in EDTA-coated tubes to prevent coagulation, and downstream depletion of red blood cells was performed by different approaches using various combinations of ACK lysis buffer, Histopaque-1119, anti-Ter119 microbeads and globin mRNA blockers (Fig 5a–5c). Magnetic microbeads coated with antibodies binding Ter119, a lineage marker for mature murine erythroid cells and their precursors, allows for negative selection of the cells of interest. Combinations including this method resulted in enriched leukocyte populations, as shown by single-cell RNA-sequencing (Fig 5a and 5b, S7–S9 Figs), with minimal red blood cell debris contamination (Fig 5b and 5c, range: 1.3%–5.2%) and sample viability exceeding 95% (97.66% ± 1.77 SD) (S2 Table). In comparison, the singular use of ACK lysis consistently incompletely removed all RBCs (19.2% contamination for ACK) and had a negative impact on the lymphocyte viability (S10 Fig, S3 Table). Combination of both ACK lysis and Histopaque purification still retrieved samples with red blood cell contamination in high percentages (29.7%) while the cleanest samples were obtained by the combined anti-Ter119 microbeads negative selection with mRNA blockers (Fig 5a–5c, Table 2, 1.3% contamination). A commercially available protocol was customized to be compatible with the 10X Genomics workflow, allowing for the blockers to bind the globin mRNA at the single-cell level (inside the droplets). Using this combination, we were able to recover and detect all the different types of leukocytes with excellent viability, while minimizing red blood cell contamination.

**Table 2:** Cell number distribution of the difference cell populations per blood isolation protocol. The aggregated sum per cell type for each protocol is shown, an overview of the cell type annotations can be found in S9 Fig.

	ACK	Histopaque1119	Histopaque1119+ACK	Microbeads+ACK	Microbeads+ACK+Blockers	Microbeads+Blockers
**B**	918	251	539	9585	16479	6205
**Basophils**	65	65	16	73	133	72
**CD4+Naive**	1722	120	872	1293	3305	1821
**CD4+Tregs**	320	23	135	247	329	379
**CD8+Memory**	568	135	268	706	1245	645
**CD8+Naive**	1260	36	492	1622	3834	1647
**cDC**	51	205	11	51	120	38
**Monocytes**	254	36	97	643	1545	541
**Neutrophils**	557	1	51	585	1397	689
**NK**	299	0	207	237	900	195
**Platelets**	33	126	23	49	70	117
**RBC_LowQuality**	1441	14439	1146	394	1608	163
***Total***	7488	15437	3857	15485	30965	12512

## Discussion

In order to systematically investigate both the liver microenvironment and the peripheral landscape in the context of liver disease, a reproducible and robust protocol was designed to retrieve high quality cells from both compartments. The protocol optimization was performed in the context of hepatitis B virus, where the immune competent AAV-HBV mouse model is used to preclinically test novel therapeutic strategies [14,15]. With the results obtained from the different procedures tested, a final protocol was established that ensures high cell quality, preserves cell proportions and cell type diversity from both liver and blood.

**Fig 6:**
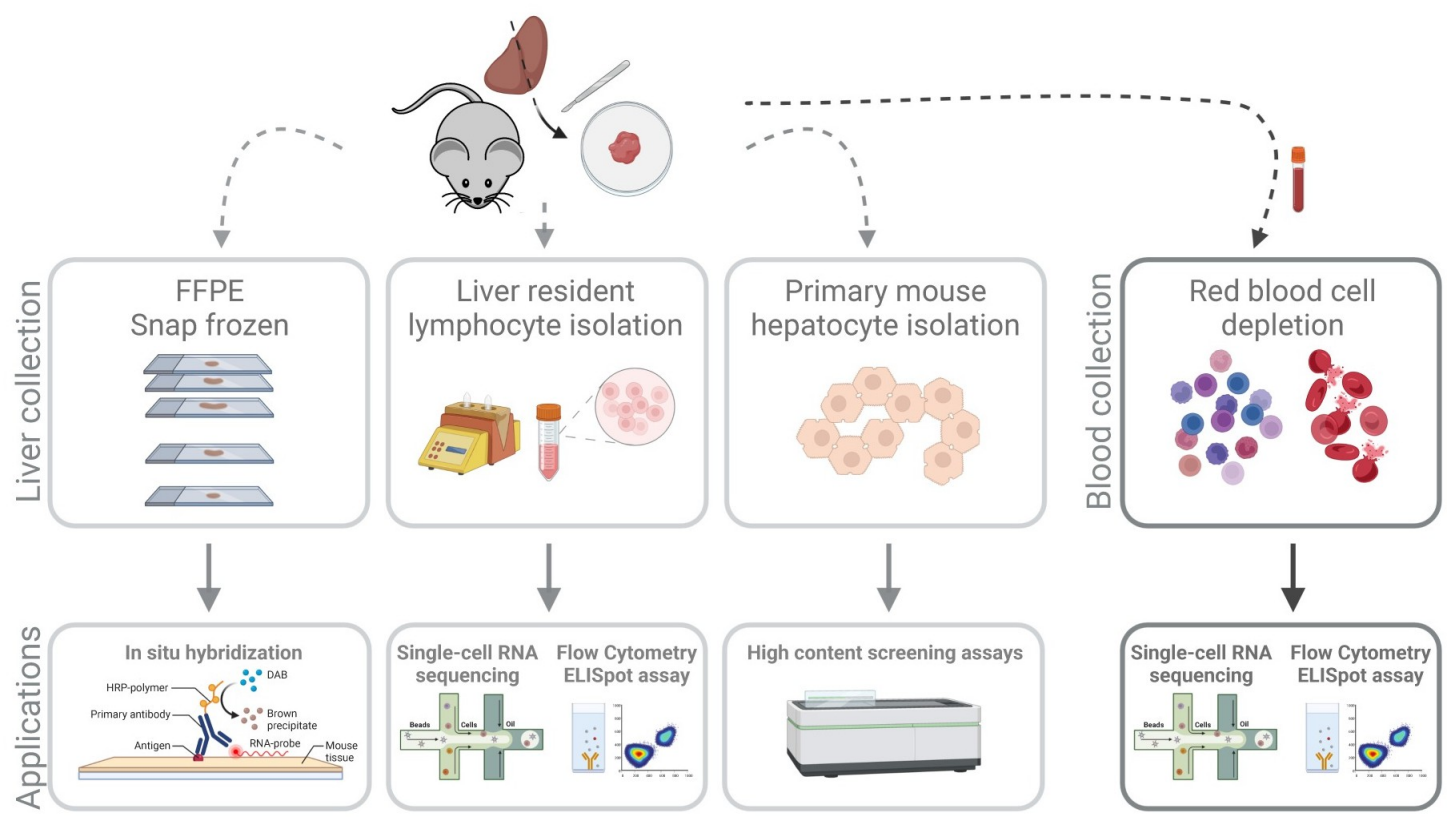
Overview of possible downstream applications after isolation of mouse liver and blood cells.

This protocol characterized the major cell populations in the liver by extracting both hepatocytes and non-parenchymal cells with high viability using only the left lateral liver lobe and allowed for the remaining lobes to be used for alternative purposes to maximize the data set that could be collected from a single animal. As the NPCs were now more abundantly extracted, it allowed to initiate more than one assay from a single sample, such as multicolor staining panels for flow cytometry, antigen recall testing of lymphocytes by using IFN-γ ELISpot or RNA sequencing. The isolated primary mouse hepatocytes were amenable to cultivation, *in vitro* compound testing and, for a limited time, cryopreservation (Fig 6). The surplus liver tissue can be stored as FFPE blocks to perform immunohistochemistry or RNAscope [16,17] and can be homogenized for RT-qPCR or they can be snap frozen for spatial transcriptomics to assess gene expression in several cell population by using 10X single-cell RNA-sequencing. Although for single-cell RNA-sequencing, only 20,000 cells are typically used as input (to balance cost and doublet rates), for other assays a more substantial input is required to obtain robust data (e.g., multicolor flow cytometry). If insufficient cell numbers are a concern, digesting several lobes of the liver in parallel will increase cell yield, but may sacrifice the availability for other readouts. This flexibility in combination with a rational study design could however keep the number of animals per study to a minimum whilst retaining the ability to scale seamlessly when needed.

Kupffer cells are the major orchestrators of immune responses and regulation in the liver, phagocytosing various antigens and particulates originating from the gastro-intestinal tract and the circulation [18–20]. Their location in the sinusoidal space (Fig 2) and close association with LSEC lining, with whom they share expression of many cell adhesion molecules [21], make it challenging to isolate them. Interestingly, only with enzymatic disintegration of the cellular matrices of the liver, both cell types could be recovered (Fig 3). We speculate that either the pronounced homo- and heterotypic cellular interactions can mechanically stress the cells beyond their structural tolerances and potentially lead to their disintegration [22]. Alternatively, incomplete dissociation of the tissue could result in the formation of large multicellular aggregates of tissue-embedded cells which are simply withheld by straining the dissociates early in the purification protocol. Of note, cholangiocytes and other immune cells with a complex phenotype (cDC and stellate cells) were also only detected after an enzymatic treatment of the liver tissue. We cannot exclude that these cell types are not picked up in the mechanical sample dissociation due to low cell numbers of cells captured in this protocol using single-cell RNA-sequencing (S2 Fig), which cannot be the case for the LSECs and Kupffer cells, which make up the majority of cell types detected in the liver.

Single-cell transcriptomics is a powerful tool to unravel the complexity of cell subpopulations and investigate the phenotype of the rare ones. However, to study leukocytes subpopulations, the large number of red blood cells present in liver as well as in whole blood, together with the need of isolating enough viable cells, signifies a big challenge for the single-cell transcriptomic approach.

Our studies required optimizing a protocol for mouse samples that would capture granulocytes and other leukocyte populations, while excluding the red blood cells as erythrocytes are known to impact both ELISpot and single-cell RNA-sequencing readout quality [23]. The depletion of red blood cells was necessary to avoid underestimation of cell concentration loaded into the microfluidic chip and avoid capture of red blood cells during droplet formation. Furthermore, the globin mRNAs still present in red blood cells are easily amplified at the expense of the mRNA from the cells of interest, leading to a massive contamination easily detectable at cDNA profile and at sequencing level [24]. Therefore, this would lead to higher sequencing costs and to increased challenges in downstream data analysis. Sampling peripheral blood in both human and mouse species is a convenient way of monitoring disease states and responses to therapy, but to obtain high quality leukocytes in sufficient number from mouse models poses some unique challenges. In conventional protocols, pre-formulated density-separation reagents for human blood are not optimized for the physical properties of murine blood and other specialty buffers do not have the expected separating power to completely exclude red blood cells from the leukocytes [25]. The tested blood purification protocols showed various performance, with the ACK lysis and Histopaque 1119 density gradient not being able to remove all red blood cells efficiently. The anti-Ter119 microbeads purification yielded the best results, which were further improved with an addition of mRNA blockers, a pool of Locked Nucleic Acid (LNA)-enhanced oligonucleotides used as a novel method to remove highly abundant RNA with low scientific value from RNA-sequencing libraries and enrich mRNA from whole blood [26,27]. Thusly, we describe an optimized method to remove red blood cells from peripheral blood without compromising sample integrity for 10x single-cell RNA-sequencing or for *ad interim* immunogenicity studies such as IFN-γ ELISpot.

It is recommended that immunogenicity studies and flow cytometry of IHLs are performed on fresh material due to the low number of cells typically isolated. We did not explicitly assess the impact on cell viability and the possible loss of certain cell populations after cryopreservation, however, a dramatic increase in handling steps will likely result in decreased recovery of material. In contrast to the IHLs, the hepatocytes can be cryopreserved for a limited period before using them *in vitro*. The freezing procedure is a crucial step in this process and further development of an optimal protocol is key to banking these cells long-term. Despite these challenges, we were able to treat thawed primary mouse hepatocytes with a capsid assembly modulator (CAM-A) causing aggregation of aberrant HBV capsids (speckles) and interfering with HBsAg production, as previously was shown by our team and others [28–30].

Taken together, the optimized mouse blood protocol described in this work opens up the possibility to longitudinally study cohorts of animals by periodically sampling small volumes of blood, determining both changes in the viral components using plasma and evolution of the cellular compartment due to the chronic hepatitis and/or the effects of administered treatments. Although liver biopsies in the form of fine needle aspirates are now becoming more common in NASH and chronic HBV/HCV patients, liver core biopsies are still not easily obtained and hematological investigations thus remain a very convenient tool for both researchers and clinicians to gain insight into disease progression.

## Materials and methods

The cell isolation protocols described in this peer-reviewed article are published on protocols.io (https://dx.doi.org/10.17504/protocols.io.81wgbzpz3gpk/v1) and are included for printing purposes as S1 File. Other routine protocols used to generate proof-of-concept data are listed below.

### Animal models

Female C57BL/6 mice were transduced with 3x10^10^ viral genome equivalents (vge) of rAAV8-HBV1.3-mer WT replicon (BrainVTA, Wuhan, China) per mouse via the tail vein. Mice were sacrificed once persistent HBV viremia was reached (day 28 after transduction). Blood sampling was performed on weekly base to assess HBsAg levels in serum. Uninfected mice were used as control.

### Liver dissociation methods

#### Mechanical liver dissociation

Mouse liver was perfused with 20 mL PBS via the hepatic portal vein. Perfused liver was carefully dissected and transferred to a C tube (Miltenyi Biotec) containing 9 mL RPMI. Tissue dissociation was done using program ‘m_spleen_04’ of the GentleMACS Octo Dissociator (Miltenyi Biotec). Liver single cell suspension was stored on ice until further processing.

#### Enzymatic liver dissociation

Primary Mouse Hepatocytes (PMH) and Non-Parenchymal Cells (NPC) were obtained by perfusing the left liver lobe using a GentleMACS Octo Dissociator with heaters and the Liver Perfusion Kit for mouse according to the manufacturer’s protocol (Miltenyi Biotec). Briefly, the left lateral lobe from mouse liver was carefully dissected and inserted in the GentleMACS Perfuser (Miltenyi Biotec). Subsequent perfusion, enzymatic digestion and dissociation of the liver lobe were performed with the buffers included in the kit according to the manufacturer’s protocol. Next, the cell suspension containing the hepatocytes and Non-Parenchymal Cells (NPC) was filtered on a MACS SmartStrainer (100 μm) (Miltenyi Biotec) and washed with DMEM + 10% FBS. Cells were stored on ice until further purification.

### Flow cytometry

IHLs were stained at 1 x 10^6^ cells/well in a 96-well U-bottom plate. All washing steps occur with 200 μL/well and centrifugation steps are set at 400x *g* for 4 minutes, room temperature. Cells are washed with PBS before eFluor780 fixable viability stain (eBioscience) was added for an incubation period of 30 minutes, 4°C. After washing with stain buffer. Fc receptors-mediated binding of antibodies is reduced by blocking with Mouse BD Fc Block (1 μL/sample, BD) and non-specific binding of antibodies and fluorophores to monocytes and macrophages is reduced by using True-Stain monocyte blocker (5 μL/sample, BioLegend). Cells are incubated with a mix of both for 10 minutes, 4°C. After the blocking steps, cells are stained for 30 minutes, 4°C, with an antibody mix (S4 Table). The cells are washed twice with stain buffer and fixated with Cytofix/Cytoperm (BD). Finally, they are washed again two times with staining buffer. Read out was done using LSRFortessa flow cytometer (BD Biosciences). Analysis was performed using FlowJo (BD).

#### Flow cytometry panel design guidelines

Liver tissue dissociation by this protocol is a combination of gentle mechanical disruption and enzymatic digestion of the extracellular matrix. When designing a multicolor flow panel, it needs to be considered that the integrity of some cell surface epitopes can be affected due to the presence of certain amino-acid sequences that can be targeted by the enzyme mix. A comprehensive list of (un)affected immune cell markers is provided by the manufacturer, but sensitivity to the mix of any novel (bio)marker of interest should be validated beforehand.

### Single-cell RNA-sequencing data analysis of liver and whole blood

Cells were processed according to the 10x Chromium Single Cell V(D)J Reagent Kits User Guide and loaded on to the Chromium 10x platform using the 5’ v2 chemistry (10x Genomics). Libraries were sequenced on a NovaSeq6000 platform (PE150) (Illumina) to an average of 50,000 reads per cell. Read alignment was done using the Cell Ranger software (10x Genomics) against the mouse transcriptome reference (mm10). Resultant cell by gene matrices for each sample were merged across all conditions tested and samples. Pre-processing, alignment, and data filtering were applied equivalently to all samples using internally implemented OpenPipeline v0.7.0 workflows. Cells with less than 1,000 Unique Molecular Identifiers (UMIs) or less than 200 genes or more than 25% mitochondrial counts were removed from downstream analysis. All downstream analysis was done in R v4.0.5 [31] using the Seurat v4.1.1 package [32].

Data was log-normalized with a scaling factor of 10,000. The top 2,000 most variable genes as determined by the ‘vst’ method implemented as the FindVariableFeatures() function were selected and scaled using a linear model implemented as the ScaleData() function. After principal component analysis (PCA) was run, the number of significant principal components (PCs) to be used for downstream cell clustering was determined using an ElbowPlot and heatmaps. The best resolution for clustering was determined using an average silhouette scoring across all clusters, testing 10 resolutions between 0.1 and 1 as previously implemented in Ziegler *et al* [33]. Marker genes for each cluster were calculated using the FindAllMarkers() function (method = ‘wilcox’) implemented in Seurat. Clusters were annotated as cell type populations based on the expression of genes that are known markers of specific cells.

### Ex vivo culturing of primary mouse hepatocytes

Freshly isolated mouse hepatocytes were diluted to 2.5 x 10^5^ cells/ml in UPCM (In Vitro ADMET Laboratories, USA) and 2.5 x 10^4^ mouse hepatocytes were seeded per well of a 96-well Biocoat collagen-coated plate. After 5 hours, when cells have attached, the plating medium was replaced with Williams E medium (Life Technologies) supplemented with primary hepatocyte maintenance supplements (Life Technologies) and a range of concentrations of a capsid assembly modulator (CAM-A) in 2% DMSO to facilitate HBV replication and protein expression. Hepatocytes were incubated at 37°C in a humidified 5% CO_2_ atmosphere while the hepatocyte medium (supplemented Williams E medium containing varying doses of a capsid assembly modulator in 2% DMSO) was replaced every 3–4 days for the duration of the experiment (up to 14 days).

### HBsAg CLIA

HBsAg secretion in the supernatant was quantified by CLIA (Autobio Diagnostics, #CL0310-2) using manufacturer’s instructions. Briefly, 50 μL of standards or samples were added into the detection plate and 50 μL of anti-HBs-Enzyme conjugate was added per well. After 1 hour incubation at 37°C, the plate was washed 6 times with wash buffer. Next, 50 μL of chemiluminescence substrate was added and incubated for 10 minutes in the dark. The chemiluminescence was measured by a Viewlux instrument (Perkin Elmer). Each plate was individually analyzed, and values were back calculated to IU/ml using the individual plate CLIA standard and expressed as % inhibition to vehicle control.

### Imaging

Plated mouse hepatocytes were fixed with 5% ultra-pure methanol-free formaldehyde (Polysciences) and permeabilized using 0.5% Triton-X-100 (Sigma-Aldrich) dissolved in PBS. Core protein was detected with primary monoclonal mouse anti-HBV core antibody (Abcam, UK). HBsAg was detected with primary recombinant human anti-HBsAg antibody (antibody generated in-house). After overnight incubation at 4°C, cells were washed three times with PBS and primary antibody was detected with Alexa Fluor-488 goat anti-mouse antibody (Thermo Fisher Scientific) or CF568 goat anti-human antibody (Biotium) for core and HBsAg respectively. Nuclei were stained using Hoechst 33258 (Thermo Fisher Scientific). Automated imaging was performed using CV8000 high-content screening system (Yokogawa). Capsid assembly modulator experiments were performed as previously published [34,35]. Brightfield images were acquired on a Revolution instrument (Echo).

## Ethics declaration

### Animal experimentation

Animal experiments were conducted in strict accordance with the guidelines established by the Institutional Animal Care and Use Committee of Janssen Pharmaceutica N.V. and complied with the ARRIVE guidelines. The local Johnson & Johnson Ethical Committee approved all experimental protocols under project license number ‘Proj 037_02-AAV HBV / HDV’, and the actual experiments were carried out following the guidelines of the European Community Council directive of 24 November 1986 (Declaration of Helsinki 86/609/EEC). The mice were maintained and handled in AAALAC accredited animal facilities following institutional and national guidelines and regulations. All efforts were made to minimize animal discomfort and to limit the number of animals used. All mice used in these experiments were female C57BL/6 mice (8–12 weeks old) obtained from Janvier (Le Genest-St-Isle, France).

## Supporting information

S1 Fig Counts of NPCs. (a) Counts after mechanical versus enzymatic digestion and (b) image of the wells just before starting flow cytometry staining (MILT = enzymatic method, MECH = mechanical disruption).
(TIF)

S2 Fig Cell counts from the single-cell RNA sequencing liver method comparison. (PDF)

S3 Fig Violin plot with the number of genes, transcripts and mitochondrial content in the two liver isolations. (PDF)

S4 Fig Box plot proportion of the different cell types across the two liver protocols. An overview of the cell type annotations can be found in S5 Fig.
(PDF)

S5 Fig Dendrogram with the cell type annotation from the liver cell populations across the two methods. (PDF)

S6 Fig Mouse hepatocytes that were thawed 5 weeks post-freezing and plated (a) showed morphological disturbance compared to fresh plated cells. Images were taken with Echo revolution microscope at day 1 and day 14. (b) Inconsistent inhibition of HBsAg in the supernatant of the hepatocytes culture.
(PDF)

S7 Fig Cell counts from the single-cell RNA sequencing of blood after different purification methods. (PDF)

S8 Fig Violin plot with the number of genes, transcripts and mitochondrial content in the four blood purification protocols. (PDF)

S9 Fig Dendrogram with the cell type annotation from the blood cell populations across the different purification methods. (PDF)

S10 Fig Cellcounter AO/PI fluorescence images illustrating red blood cell contamination after different isolation protocols. (a), (d) are Histopaque isolation; (b), (c) are ACK+Histopaque isolation; (e), (h) are ACK isolation; (I) is anti-Ter Microbeads isolation; (l), (n) are ACK+anti-Ter Microbeads isolation. All the images were acquired on a Luna FX7 Automated Cell Counter (Logos biosystem).
(PDF)

S1 Table Viability of hepatocytes before freezing and after thawing after 1 versus 5 weeks of storage in liquid nitrogen vapor phase. (XLSX)

S2 Table Overview of cell count and viability of 40 blood samples after RBC removal using the procedure with anti-Ter119 beads. (XLSX)

S3 Table Overview of all blood samples profiled using single-cell RNA sequencing. (PDF)

S4 Table Flow cytometry antibody panel and panel design guidelines. (PDF)

S1 File Step-by-step protocol, mechanical liver dissociation, also available on protocols.io. (PDF)

S2 File Step-by-step protocol, enzymatic liver dissociation, also available on protocols.io. (PDF)

S3 File Step-by-step protocol, ex vivo cell isolation, also available on protocols.io. (PDF)

S4 File Step-by-step protocol, blood sampling, cell isolation, and sequencing library preparation, also available on protocols.io. (PDF)
