# Supplementary figures and images for: Robust isolation protocol for mouse leukocytes from blood and liver resident cells for immunology research
Source: PLoS One. 2024 Aug 22;19(8):e0304063. doi: 10.1371/journal.pone.0304063 (PMC11340898; doi:10.1371/journal.pone.0304063)

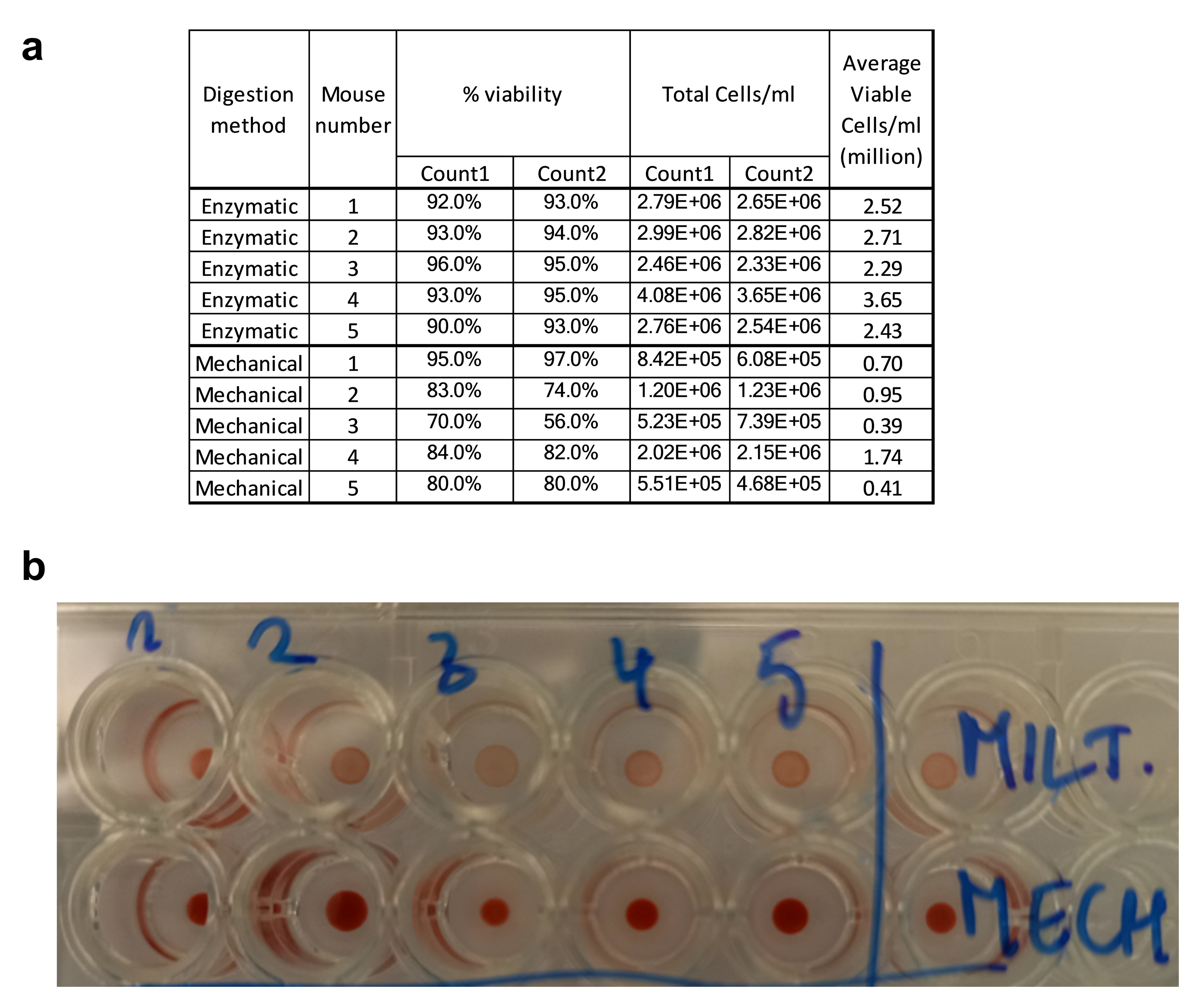

Supplement: S1 Fig — (a) Counts after mechanical versus enzymatic digestion and (b) image of the wells just before starting flow cytometry staining (MILT = enzymatic method, MECH = mechanical disruption). (TIF) [file pone.0304063.s001.tif]

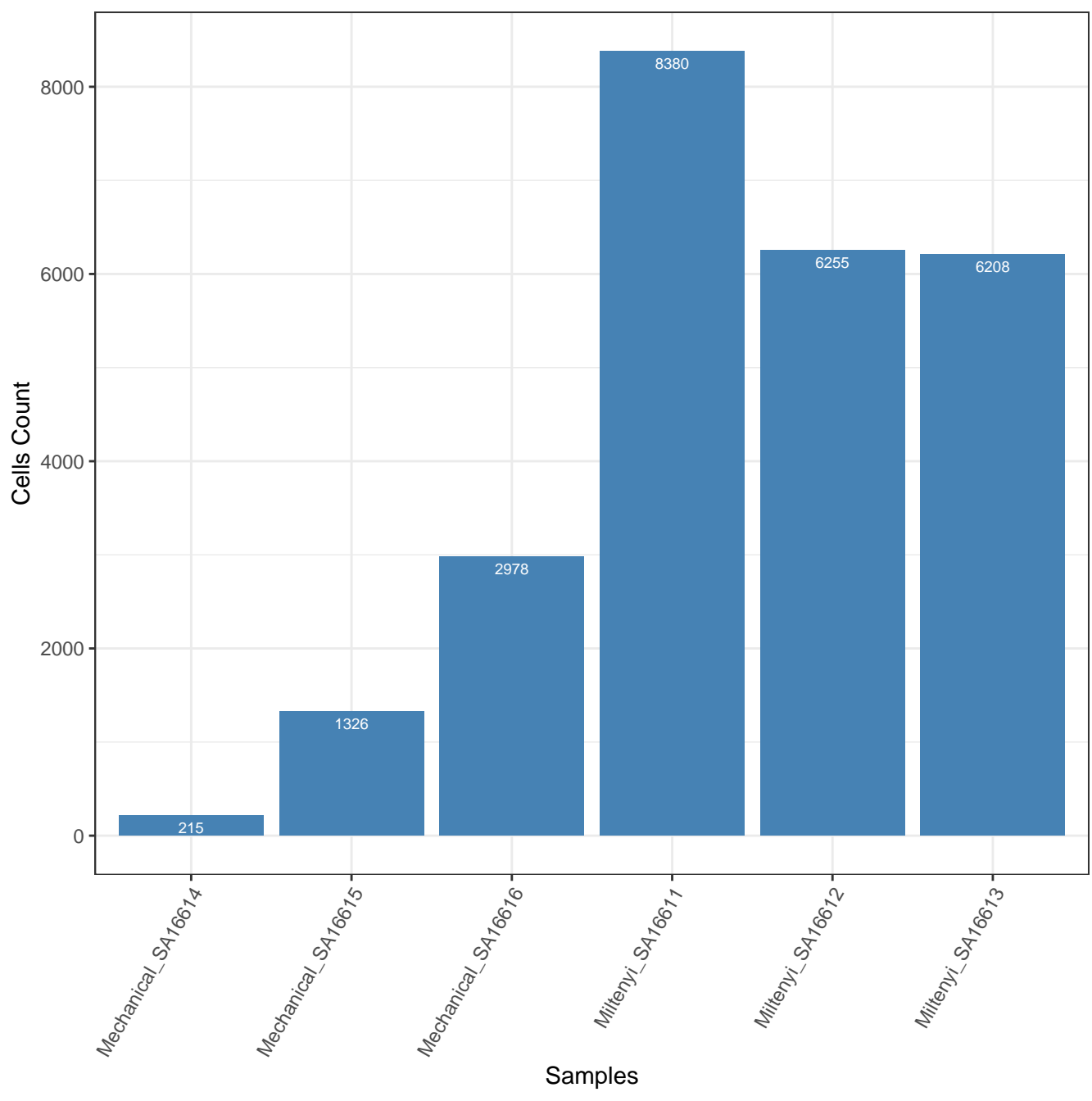

Supplement: S2 Fig — (PDF) [file pone.0304063.s002.pdf]

**n\_genes**

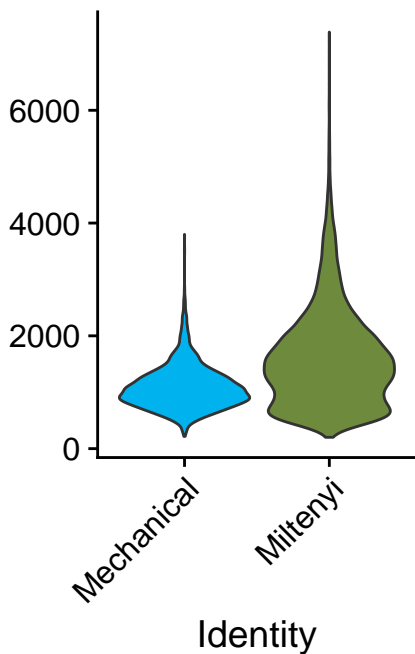

**n\_counts**

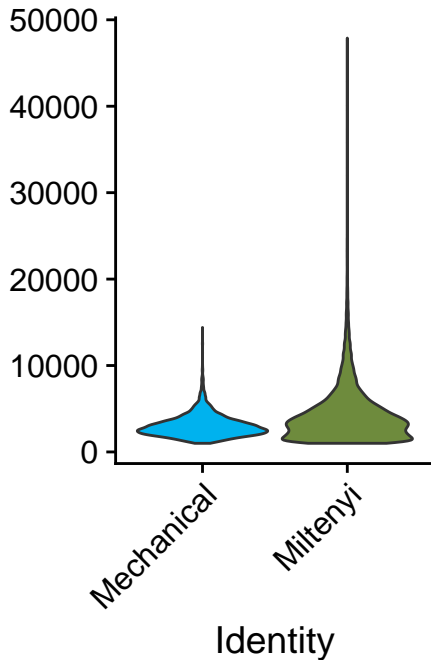

**percent\_mito**

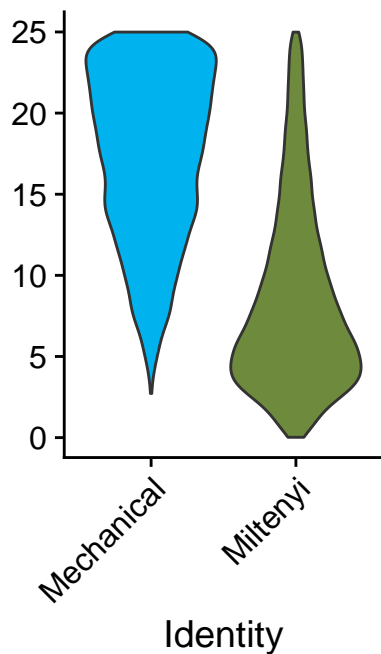

Supplement: S3 Fig — (PDF) [file pone.0304063.s003.pdf]

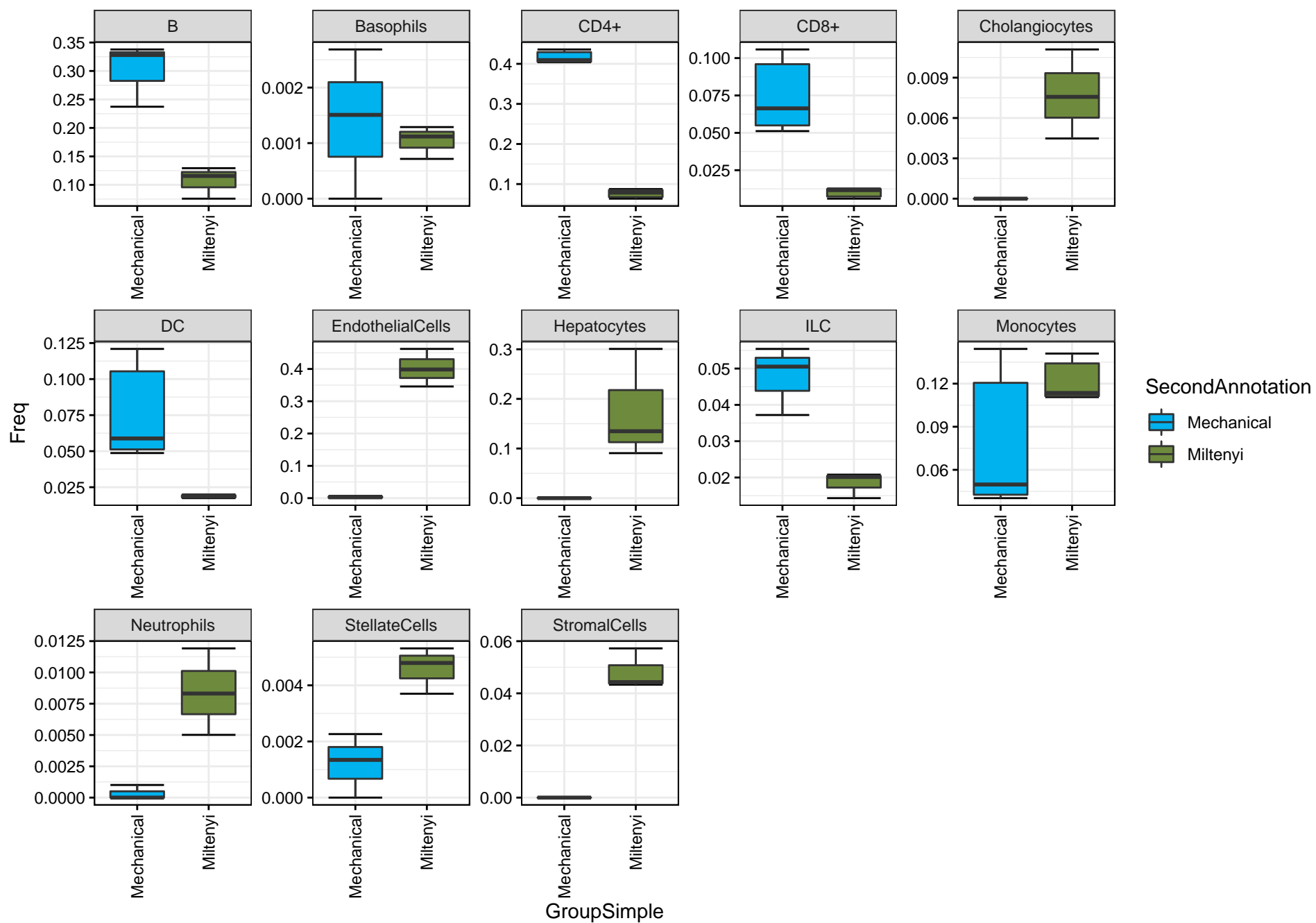

Supplement: S4 Fig — An overview of the cell type annotations can be found in S5 Fig. (PDF) [file pone.0304063.s004.pdf]

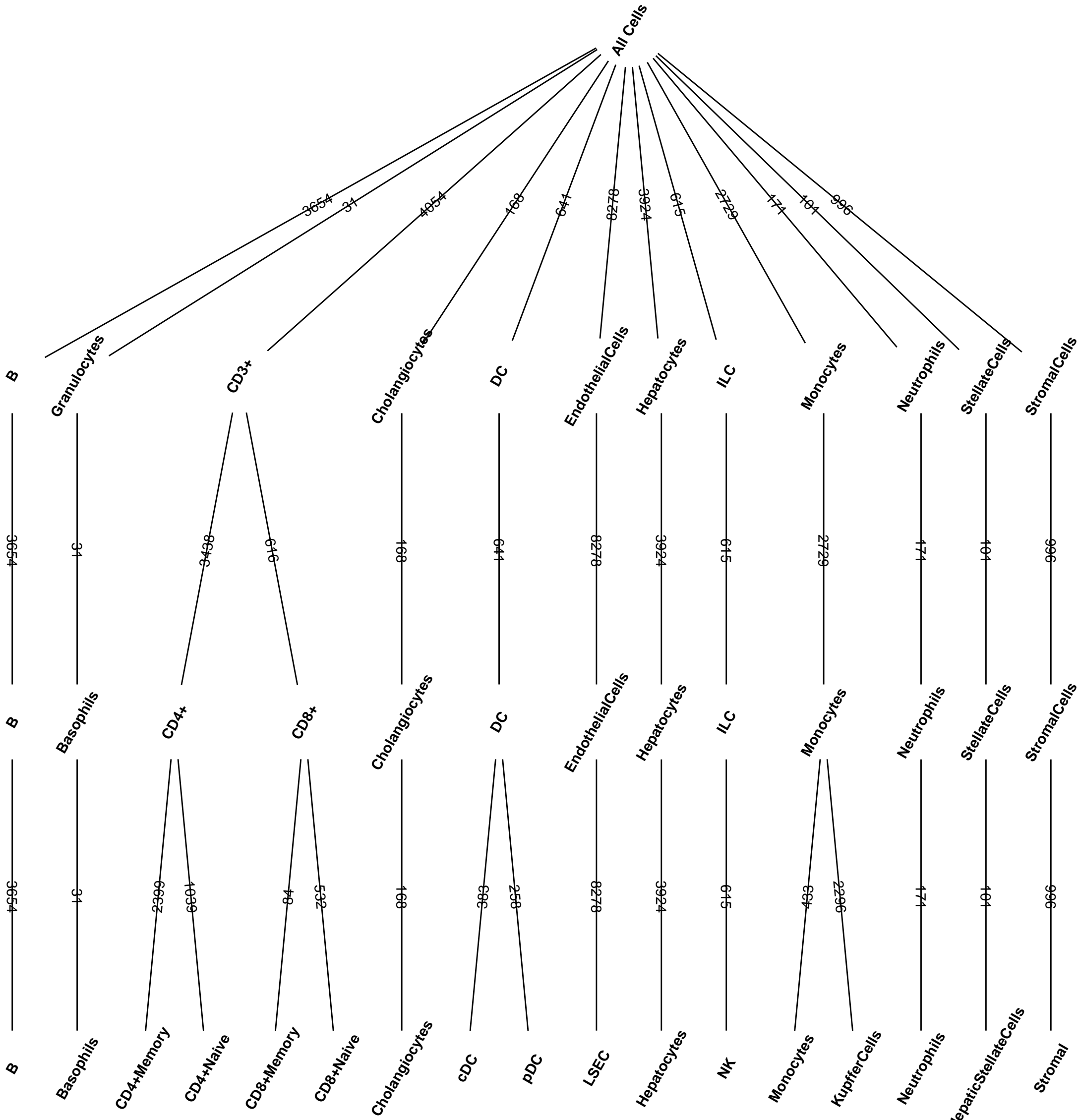

Supplement: S5 Fig — (PDF) [file pone.0304063.s005.pdf]

**a**

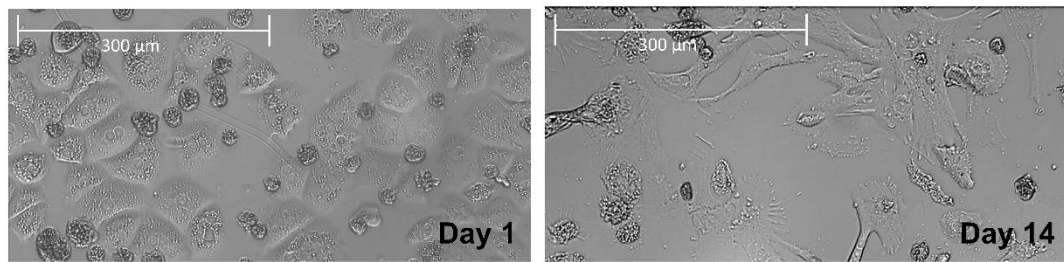

**b**

**Secreted HBsAg  
of hepatocytes thawed after 5 weeks**

**Day 14**

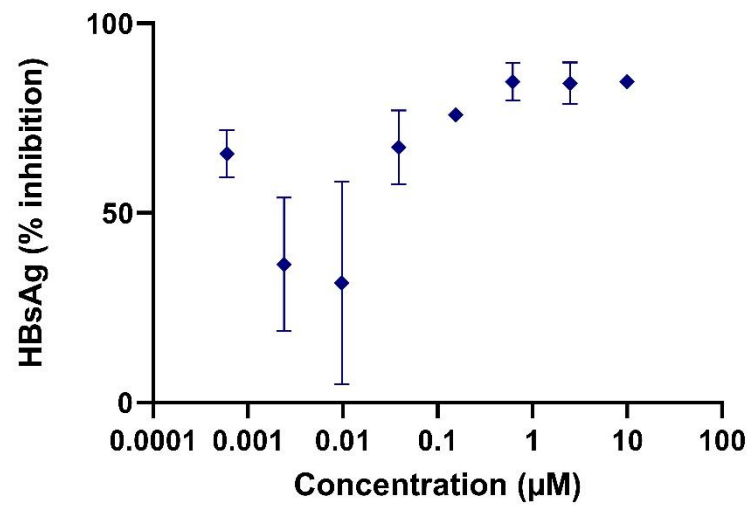

Supplement: S6 Fig — Mouse hepatocytes that were thawed 5 weeks post-freezing and plated (a) showed morphological disturbance compared to fresh plated cells. Images were taken with Echo revolution microscope at day 1 and day 14. (b) Inconsistent inhibition of HBsAg in the supernatant of the hepatocytes culture. (PDF) [file pone.0304063.s006.pdf]

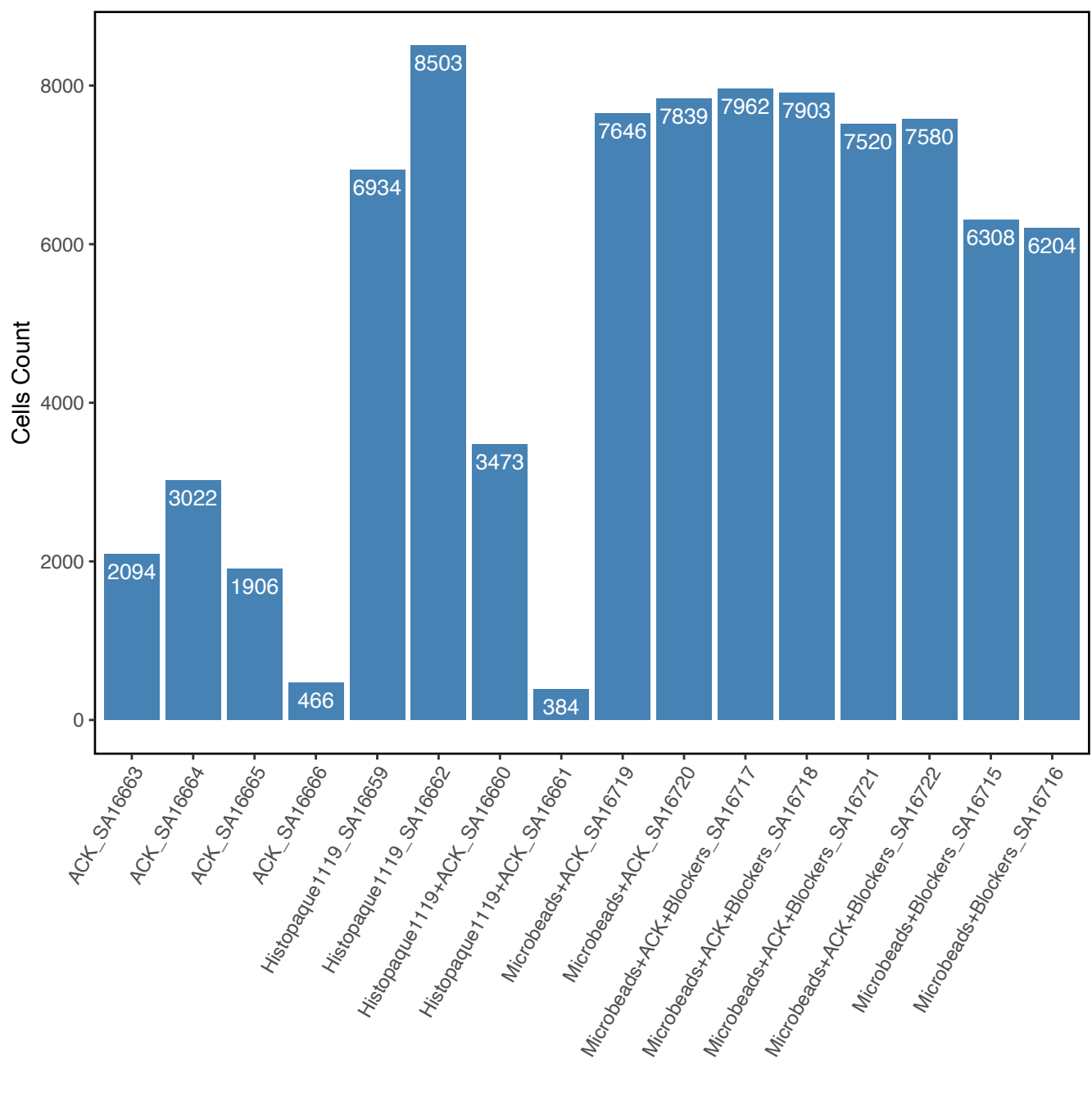

Supplement: S7 Fig — (PDF) [file pone.0304063.s007.pdf]

**n\_genes**

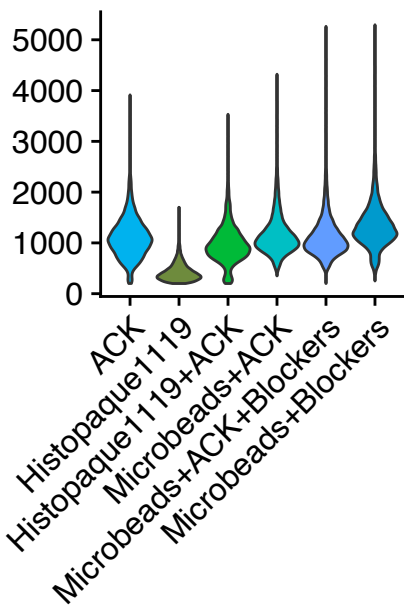

**n\_counts**

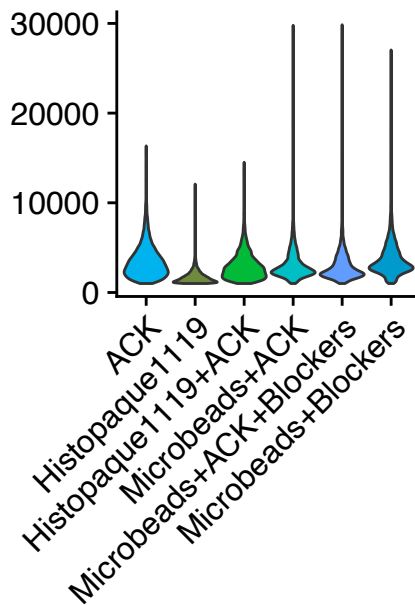

**percent\_mito**

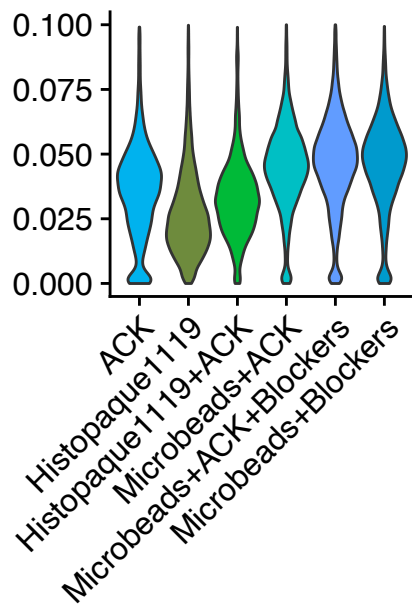

Supplement: S8 Fig — (PDF) [file pone.0304063.s008.pdf]

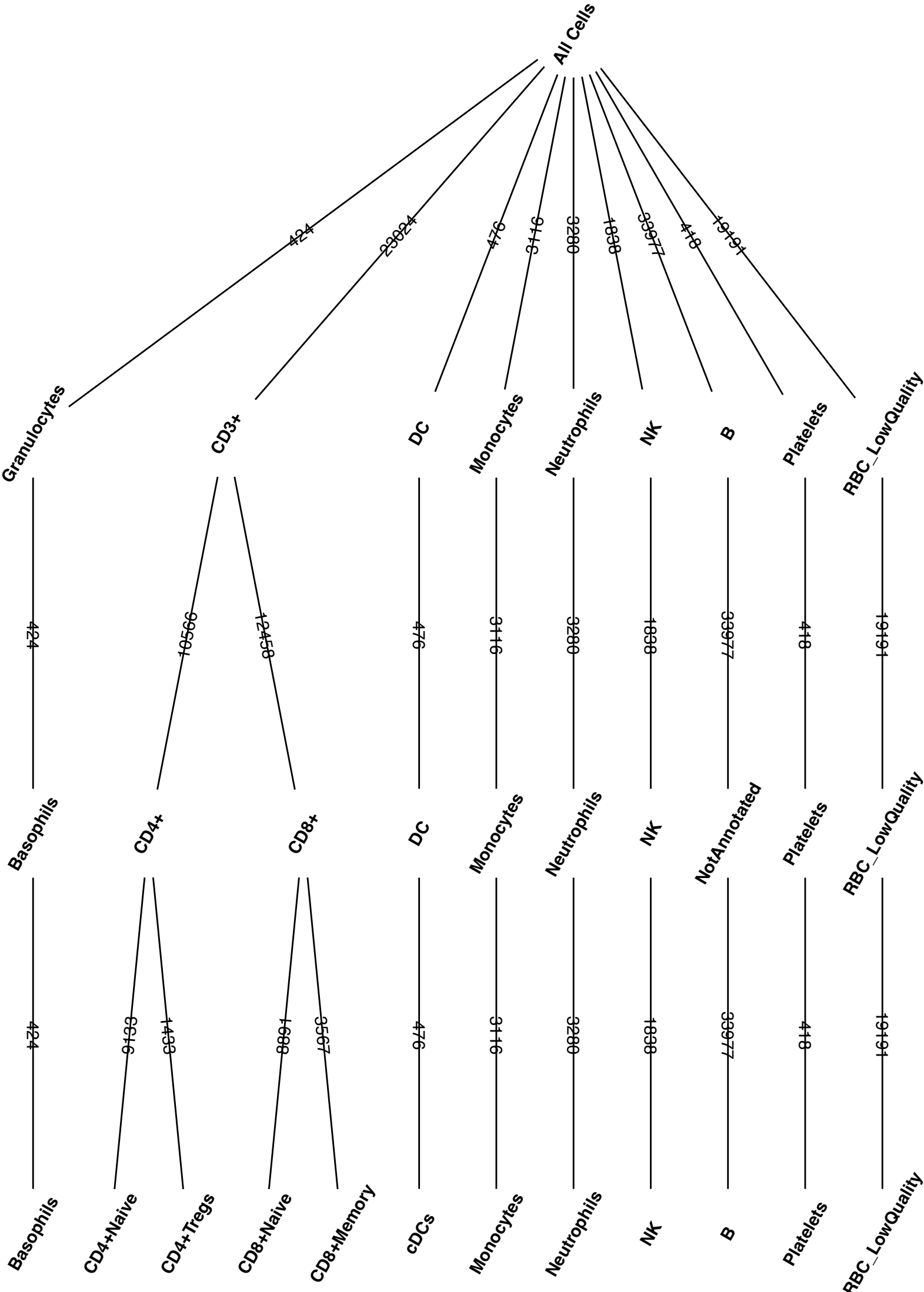

Supplement: S9 Fig — (PDF) [file pone.0304063.s009.pdf]

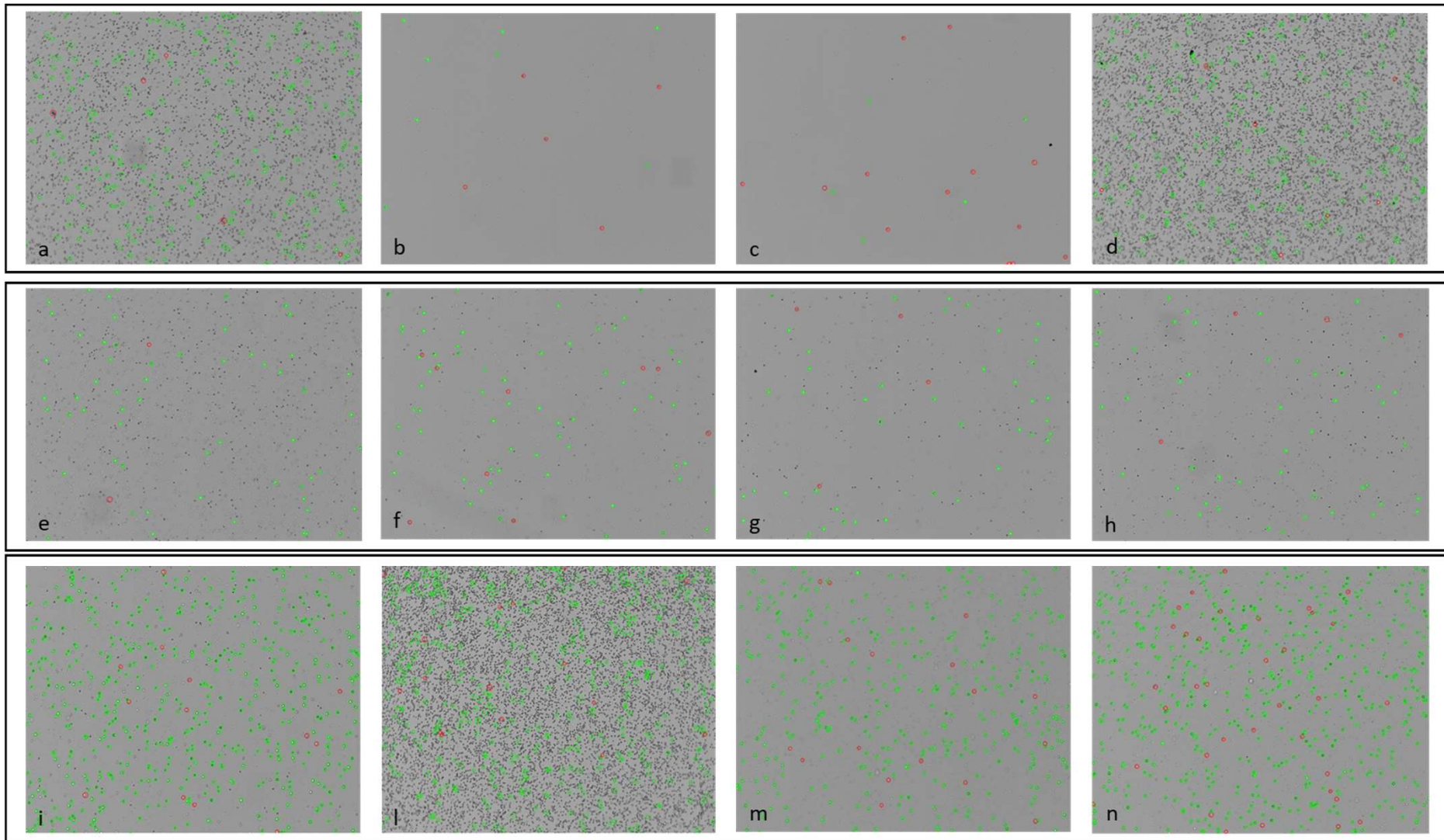

Supplement: S10 Fig — (a), (d) are Histopaque isolation; (b), (c) are ACK+Histopaque isolation; (e), (h) are ACK isolation; (I) is anti-Ter Microbeads isolation; (l), (n) are ACK+anti-Ter Microbeads isolation. All the images were acquired on a Luna FX7 Automated Cell Counter (Logos biosystem). (PDF) [file pone.0304063.s010.pdf]
