# Supplementary material for: Robust isolation protocol for mouse leukocytes from blood and liver resident cells for immunology research
Source: PLoS One. 2024 Aug 22;19(8):e0304063. doi: 10.1371/journal.pone.0304063 (PMC11340898; doi:10.1371/journal.pone.0304063)
Supplement: S3 Table — (PDF) [file pone.0304063.s013.pdf]

Sample ID	isolation protocol	mRNA blockers	viability	cell count tot (cell/ml)	cell count live (cell/ml)	cell count dead (cell/ml)	notes
**SA16659**	Histopaque1119	no	96.80%	$2.62*10^6$	$2.54*10^6$	$8.46*10^4$	
**SA16660**	ACK+Histopaque1119	no	50.70%	$1.58*10^5$	$8.02*10^4$	$7.80*10^4$	plus ACK step
**SA16661**	ACK+Histopaque1119	no	36.00%	$1.67*10^5$	$6.01*10^4$	$1.07*10^5$	plus ACK step
**SA16662**	Histopaque1119	no	97.40%	$2.59*10^6$	$2.52*10^6$	$6.68*10^4$	
**SA16663**	ACK	no	95.40%	$7.28*10^5$	$6.95*10^5$	$3.34*10^4$	
**SA16664**	ACK	no	86.90%	$7.48*10^5$	$6.50*10^5$	$9.80*10^4$	
**SA16665**	ACK	no	89.60%	$5.77*10^5$	$5.17*10^5$	$6.01*10^4$	
**SA16666**	ACK	no	90.80%	$5.08*10^5$	$4.61*10^5$	$4.68*10^4$	
**SA16715**	anti-Ter 119 Microbeads	yes	98.30%	$6.03*10^6$	$5.93*10^6$	$1.05*10^5$	
**SA16716**	anti-Ter 119 Microbeads	yes					alternative GEM-RT
**SA16717**	ACK+anti-Ter Microbeads	yes	95.80%	$6.26*10^6$	$6*10^6$	$2.61*10^5$	
**SA16718**	ACK+anti-Ter Microbeads	yes					alternative GEM-RT
**SA16719**	ACK+anti-Ter Microbeads	no	96.20%	$4.33*10^6$	$4.17*10^6$	$1.63*10^5$	
**SA16720**	ACK+anti-Ter Microbeads	no					
**SA16721**	ACK+anti-Ter Microbeads	yes	94.30%	$6.87*10^6$	$6.47*10^6$	$3.90*10^5$	
**SA16722**	ACK+anti-Ter Microbeads	yes					alternative GEM-RT
