# Supplementary material for: Robust isolation protocol for mouse leukocytes from blood and liver resident cells for immunology research
Source: PLoS One. 2024 Aug 22;19(8):e0304063. doi: 10.1371/journal.pone.0304063 (PMC11340898; doi:10.1371/journal.pone.0304063)
Supplement: S4 Table — (PDF) [file pone.0304063.s014.pdf]

Marker	Fluorochrome	Catalog Number	Supplier	Volume (µL/100µL)
CD45	BUV395	564279	BD	1
Ly6G	BV421	127628	BioLegend	2.5
F4/80	PE-Dazzle594	123146	BioLegend	2.5
Ly6C	PE-Cy7	128018	BioLegend	0.5
CD11c	PE	553802	BD	5
CD4	BV786	563331	BD	0.5
B220	PerCp-Cy5.5	561101	BD	5
CD11b	BUV496	749864	BD	2.5
CD8a	BUV737	612759	BD	5
CD49b	AlexaFluor488	108913	BioLegend	2
MHC II	APC	107614	BioLegend	0.5
CD3	AlexaFluor700	152316	BioLegend	1.25
