# Supplementary material for: Robust isolation protocol for mouse leukocytes from blood and liver resident cells for immunology research
Source: PLoS One. 2024 Aug 22;19(8):e0304063. doi: 10.1371/journal.pone.0304063 (PMC11340898; doi:10.1371/journal.pone.0304063)
Supplement: S1 File — (PDF) [file pone.0304063.s015.pdf]

Apr 12, 2024

Mechanical liver dissociation

In 1 collection

DOI
dx.doi.org/10.17504/protocols.io.8epv5r1r5g1b/v1

Dorien De Pooter[1], Ben De Clerck[1], Koen Dockx[2], Domenica De Santis[2], Sarah Sauviller[1], Pascale Dehertogh[1], Matthias Beyens[3], Isabelle Bergiers[3], Isabel Nájera[4], Ellen Van Gulck[1], Nádia Conceição-Neto[1], Wim Pierson[1]

[1]ID Discovery, Infectious Diseases Therapeutic Area, Janssen Research and Development, Beerse, Belgium;
[2]Charles River Laboratories, Beerse, Belgium;
[3]Discovery Technologies & Molecular Pharmacology, Therapeutics Discovery, Janssen Research and Development, Beerse, Belgium;
[4]ID Discovery, Infectious Diseases Therapeutic Area, Janssen Research and Development, California, Brisbane, USA

Wim Pierson
ID Discovery, Infectious Diseases Therapeutic Area, Janssen ...

OPEN ACCESS

DOI: dx.doi.org/10.17504/protocols.io.8epv5r1r5g1b/v1

Protocol Citation: Dorien De Pooter, Ben De Clerck, Koen Dockx, Domenica De Santis, Sarah Sauviller, Pascale Dehertogh, Matthias Beyens, Isabelle Bergiers, Isabel Nájera, Ellen Van Gulck, Nádia Conceição-Neto, Wim Pierson 2024. Mechanical liver dissociation. **protocols.io** https://dx.doi.org/10.17504/protocols.io.8epv5r1r5g1b/v1

License: This is an open access protocol distributed under the terms of the **Creative Commons Attribution License**, which permits unrestricted use, distribution, and reproduction in any medium, provided the original author and source are credited

Protocol status: Working

Created: April 01, 2024

Last Modified: April 12, 2024

Protocol Integer ID: 98105

Abstract

This protocol details the mechanical dissociation of liver.

Materials

Reagents:

- RPMI1640 medium with L-glutamine (Lonza, BE12-702F)
- Ethanol ≥70% (v/v) TechniSolv® **VWR International Catalog #**83801.360
- 10×PBS DPBS **Merck MilliporeSigma (Sigma-Aldrich) Catalog #**D1408

Equipment:

- gentleMACS Octo Dissociator with Heaters **Miltenyi Biotec Catalog #** 130-096-427
- Sharps container 1.5L (BD, 305624)
- 23G needle HSW HENKE-JECT (HenkeSass Wolf, 4710006025)
- GentleMACS C Tube **Miltenyi Biotec Catalog #**130-096-334

Reagent preparation

1 Add 55 mL of FCS to a bottle of 500 mL of RPMI1640 with L-Glutamine.

2 Prepare GentleMACS C-tubes by filling them with 9 mL RPMI 1640 medium + 10% FCS and store the tubes at 4 °C .

3 Fill the syringe with 20 mL PBS and connect a 23G needle to the syringe.

Procedure

4

Note

IMPORTANT! Proceed with liver perfusion shortly after euthanasia to prevent clotting of the blood.

Euthanize the mice by decapitation.

5 Position mouse in dorsal recumbency and pin down. Spray the abdominal and thoracic region with 70% EtOH.

6 Make a parallel incision from the caudal to cranial side starting from the mouse's abdomen until the thoracic region.

7 Gently move the intestines to the right side to get access to the liver using forceps and carefully flip the median lobe and left lobe to the cranial side to visualize the portal vein.

8 Insert the tip of the needle in the hepatic portal vein. Slowly push 20 mL PBS through.

Note

The liver will become tense and pale if the perfusion is correctly performed.

9 Carefully dissect the perfused liver and transfer into the C-tube containing the medium.

Note

Remove gallbladder during dissection of the mouse liver.

10 Tightly close C-tube and attach it upside down onto the GentleMACS Octo Dissociator.

11 Run program m_spleen_04 twice.

12 After termination of the program, detach C-tube from the GentleMACS Octo Dissociator and keep the tubes on ice until further processing.

13 Dispose the needle in an approved sharps container.
