# Supplementary material for: Robust isolation protocol for mouse leukocytes from blood and liver resident cells for immunology research
Source: PLoS One. 2024 Aug 22;19(8):e0304063. doi: 10.1371/journal.pone.0304063 (PMC11340898; doi:10.1371/journal.pone.0304063)
Supplement: S2 File — (PDF) [file pone.0304063.s016.pdf]

Apr 12, 2024

Enzymatic liver dissociation (with liver perfusion kit)

In 1 collection

DOI
dx.doi.org/10.17504/protocols.io.261ge5o57g47/v1

Dorien De Pooter[1], Ben De Clerck[1], Koen Dockx[2], Domenica De Santis[2], Sarah Sauviller[1], Pascale Dehertogh[1], Matthias Beyens[3], Isabelle Bergiers[3], Isabel Nájera[4], Ellen Van Gulck[1], Nádia Conceição-Neto[1], Wim Pierson[1]

[1]ID Discovery, Infectious Diseases Therapeutic Area, Janssen Research and Development, Beerse, Belgium;
[2]Charles River Laboratories, Beerse, Belgium;
[3]Discovery Technologies & Molecular Pharmacology, Therapeutics Discovery, Janssen Research and Development, Beerse, Belgium;
[4]ID Discovery, Infectious Diseases Therapeutic Area, Janssen Research and Development, California, Brisbane, USA

Wim Pierson
ID Discovery, Infectious Diseases Therapeutic Area, Janssen ...

OPEN ACCESS

DOI: dx.doi.org/10.17504/protocols.io.261ge5o57g47/v1

Protocol Citation: Dorien De Pooter, Ben De Clerck, Koen Dockx, Domenica De Santis, Sarah Sauviller, Pascale Dehertogh, Matthias Beyens, Isabelle Bergiers, Isabel Nájera, Ellen Van Gulck, Nádia Conceição-Neto, Wim Pierson 2024. Enzymatic liver dissociation (with liver perfusion kit). **protocols.io** https://dx.doi.org/10.17504/protocols.io.261ge5o57g47/v1

License: This is an open access protocol distributed under the terms of the **Creative Commons Attribution License**, which permits unrestricted use, distribution, and reproduction in any medium, provided the original author and source are credited

Protocol status: Working

Created: April 01, 2024

Last Modified: April 12, 2024

Protocol Integer ID: 98106

Abstract

This protocol details enzymatic liver dissociation.

Materials

Reagents:

- FCS, frozen (0.22µm filtered Gibco by Thermo Fischer Scientific, 011-90005M)
- DMEM(1×) + 4.5g/l D-glucose + L-glutamine DMEM, high glucose **Thermo Fisher Catalog #**41965039
- 1× Dulbecco's PBS (without calcium magnesium) **Merck MilliporeSigma (Sigma-Aldrich) Catalog #**D8537

Equipment:

- VACUSAFE aspiration system, 4L PP bottle, tubing fittings (Integra Biosciences, 391-2094)
- Microscope
- Cellaca-MX-AOPI cell counter (Nexcelom)

Materials:

- gentleMAC Perfusers **Miltenyi Biotec Catalog #**130-128-151
- gentleMACS Perfusion Sleeves **Miltenyi Biotec Catalog #**130-128-752
- GentleMACS C Tube **Miltenyi Biotec Catalog #**130-096-334
- MACS SmartStrainers (100 µm) **Miltenyi Biotec Catalog #**130-098-463
- Tissue Culture Dish (diameter 60 mm) (Falcon, 353002)
- Tissue Culture Dish (diameter 100 mm) (Falcon, 353003)

Reconstitution buffer:

A	B
Buffer P (20X)	400 µL
Sterile water	7.6 ml
Reagent C	16 µL

For 62 mL Buffer P (1x):

A	B
Buffer P (20X)	3.1 ml
Sterile distilled water	58.9 ml

Enzyme digestion mix:

A	B
Enzyme D	110 µL
Enzyme R	110 µL
Enzyme A	30 µL
Equilibration buffer	9 ml

Reagent preparation

1h 5m

1 **Storage**

Lyophilized enzymes should be stored at 2 °C – 8 °C . Buffer P (20x), Reagent C and Reagent E should be stored at Room temperature . Lyophilized enzymes should be reconstituted before the date indicated on their respective vials.

2 **Reconstitution buffer**

2.1 Prepare the reconstitution buffer by diluting 400 µL Buffer P (20×) with 7.6 mL sterile water.

A	B
Buffer P (20X)	400 µL
Sterile water	7.6 ml
Reagent C	16 µL

2.2 Add 16 µL of Reagent C and mix well.

Note

- Indicated volumes are for the reconstitution of 1 vial of lyophilized enzyme D, R and A.
- Upscale volumes accordingly if more vials need to be reconstituted.

2.3

45m

- Buffer P (20×) is susceptible to bacterial contamination. Sterile aliquoting of Buffer P (20×) is recommended.
- Store buffer P (20×) at room temperature and prewarm the buffer at 37 °C for at least 00:45:00 before first use. Shake the buffer thoroughly before use.

3 **Enzyme reconstitution**

3.1 Enzyme D

10m

- Prepare Enzyme D by reconstitution of the lyophilized powder in the vial with 3 mL reconstitution buffer. Invert the vial after closing the lid and wait 00:05:00 – 00:10:00 to dissolve the powder. Do not pipette up and down to dissolve the enzyme!

- Prepare aliquots to avoid repeated freeze-thaw cycles. Store aliquots at -20 °C .

Note

- The enzyme solution is stable for 6 months after reconstitution.
- For cell culture experiments subsequent to tissue dissociation, sterile-filter Enzyme D prior to aliquoting.
- Work on ice once the enzymes are reconstituted!
- Make sure to thoroughly mix the enzyme solution immediately before taking out the required reaction volume.

3.2 Enzyme R 10m

- Prepare Enzyme R by reconstitution of the lyophilized powder in the vial with 3 mL reconstitution buffer. Invert the vial after closing the lid and wait 00:05:00 – 00:10:00 to dissolve the powder. Do not pipette up and down to dissolve the enzyme!
- Prepare aliquots to avoid repeated freeze-thaw cycles. Store aliquots at -20 °C .

Note

- The enzyme solution is stable for 6 months after reconstitution.
- Work On ice once the enzymes are reconstituted!
- Make sure to thoroughly mix the enzyme solution immediately before taking out the required reaction volume.

3.3 Enzyme A

- Prepare Enzyme A by reconstitution of the lyophilized powder in the vial with 1 mL of reconstitution buffer. Do not vortex!
- Prepare aliquots to avoid repeated freeze-thaw-cycles. Store aliquots at -20 °C .

Note

- The enzyme solution is stable for 6 months after reconstitution.
- Work On ice once the enzymes are reconstituted!
- Make sure to thoroughly mix the enzyme immediately before taking out the required reaction volume.

4 **Perfusion buffers**

For 62 mL Buffer P (1x):

A	B
Buffer P (20X)	3.1 ml
Sterile distilled water	58.9 ml

Buffer P (1x) is used as the basis to prepare the perfusion buffers.

Note

Indicated volumes are for 1 perfusion. When performing more perfusions, upscale volumes accordingly.

5 **Pre-digestion buffer**

Transfer 44 mL of Buffer P (1x) to a new 50 mL tube.

6 **Equilibration buffer**

- Transfer 18 mL of Buffer P (1x) to a new 50 mL tube and add 36 µL Reagent C.
- Take 9 mL of the prepared equilibration buffer and transfer to a separate tube.
- This equilibration buffer will be used to prepare the enzyme digestion mix.

Note

Indicated volumes are for 1 perfusion. When performing more perfusions, upscale volumes accordingly.

7 **Enzyme digestion mix**

Prepare the enzyme digestion mix during the equilibration phase. Enzymes must be added freshly, shortly before the enzyme digestion mix is used.

A	B
Enzyme D	110 µL
Enzyme R	110 µL
Enzyme A	30 µL
Equilibration buffer	9 ml

8 **Liver Wash Buffer**

Thaw a 50 mL aliquot of FCS and prepare a solution of 9% FCS (v/v) in DMEM (1x) + 4.5g/l D-glucose + L-glutamine. This buffer can be stored at 4 °C for 3 months.

Procedure : PART I

10m

9 **A) General remarks:**

A maximum of 1.2 g of liver tissue can be perfused in one GentleMACS Perfuser.

9.1 The time from the dissection of the liver to the start of the perfusion should not exceed 00:10:00 . Longer time spans will impact cell yield and viability. 10m

9.2 The adjuster of the lid of the GentleMACS Perfuser is already provided in the correct position (original position) for the perfusion of mouse liver. Do not turn the adjuster if mouse liver will be perfused!

9.3 The position of the adjuster already altered by accident, it can be re-adjusted to the original position by turning it counterclockwise to the highest position and then slightly back until the ".5 mark" on the adjuster's scale matches the arrowhead on the adjuster.

Note

- Be careful not to accidentally turn the adjuster once the mouse liver is placed in the GentleMACS Perfuser, as needles might pierce through the liver, which will hamper the perfusion.
- If working with mouse liver, it is recommended to use the left lateral lobe, instead of the whole liver.

10 **B) Preparation:**
Ensure that the regular GentleMACS sleeves have been exchanged by the perfusion sleeves before attaching the GentleMACS perfuser.

10.1 Pre-heat water bath with beads at 37 °C .

10.2 Pre-warm pre-digestion buffer and tubes of equilibration buffer at 37 °C .

10.3 Attach the base of the GentleMACS perfuser with the lid onto the GentleMACS perfusion sleeve on the GentleMACS Octo Dissociator with heaters. Leave out the grid and the clamp.

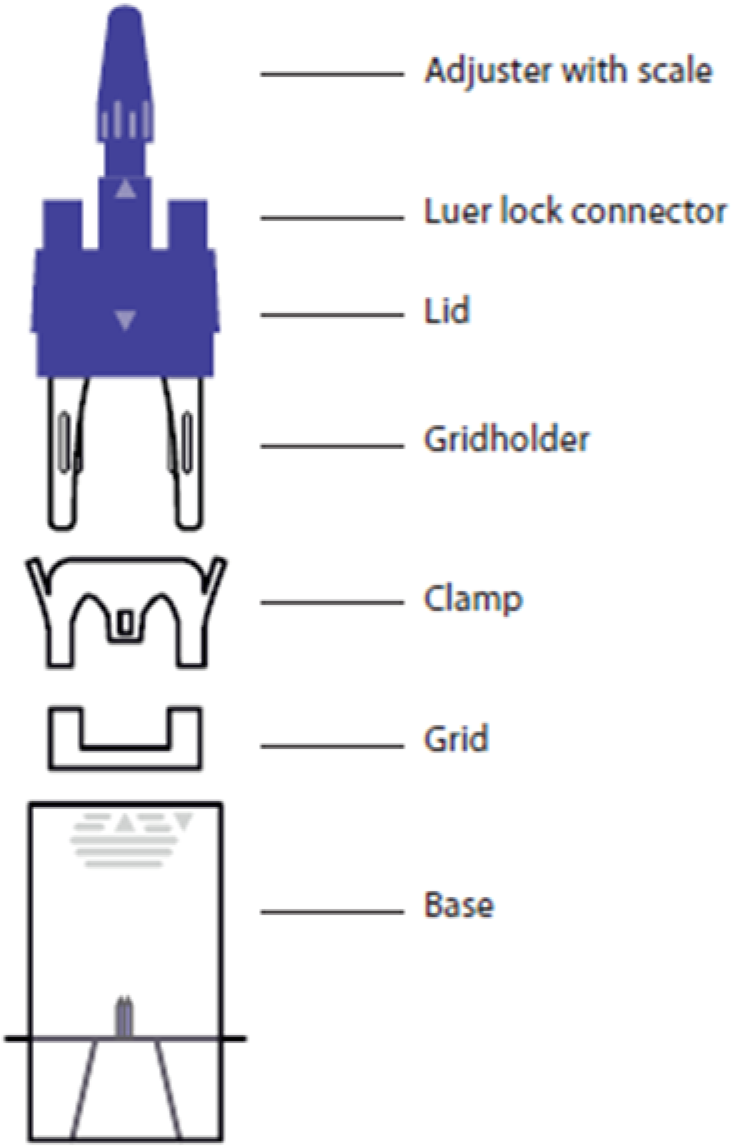

10.4 Check the adjuster in the correct position for liver tissue perfusion.

10.5 Place a heating unit onto the GentleMACS perfuser.

10.6 Carefully transfer 8 mL of warm pre-digestion buffer into the GentleMACS perfuser by using one of the two luer lock connectors.

10.7 Start the appropriate GentleMACS program for mouse 37C_m_LIPK_1.

10.8 After the priming step, the program will automatically pause and will be resumed later after the tissue sample is loaded.

10.9 Place the grid into a 100 mm tissue culture dish.

Procedure : PART II

11 **A) Mouse liver perfusion:**

11.1 Carefully dissect the left lateral liver lobe. Rinse the lobe in a 100 mm tissue culture dish filled with 1× PBS.

> **Note**
>
> Make sure that the liver lobe is not injured during dissection.

11.2 Place the liver lobe in the center of the grid.

11.3 Squeeze the clamp and attach it to the grid by pushing it down until the clamp touches the surface of the petri dish to fix the liver between grid and clamp.

> **Note**
>
> Make sure that the two channels of the grid used for buffer removal are not blocked by incorrect assembly of grid and clamp.

11.4 Remove the lid of the GentleMACS perfuser and connect it by pushing the grid holder through the clamp into the grid containing the tissue.

> **Note**
>
> A click will emerge at each side of the grid holder if the grid holder has been properly connected to the grid.

11.5 Transfer the assembly of lid-clamp-grid back onto the base of the GentleMACS perfuser.

11.6 Turn the lid slowly counterclockwise without pressure on the base until the lid drops to a lower position. Then, screw the thread of the lid clockwise without tilting to tightly close the GentleMACS Perfuser.

11.7 Resume the program which is paused after priming to continue with the initial perfusion step.

Note

No change of buffer is needed at this time since the buffer inside the GentleMACS perfuser was only used for priming the system.

11.8 After the initial perfusion step, the program will pause to start the washing phase. This phase consists of 4 cycles of buffer exchange, and the program will pause after each cycle.

Note

Follow the instructions on the display of GentleMACS instrument every time the program pauses.

11.9 During the pause, remove the used pre-digestion buffer manually with a VACUSAFE aspiration system and then add 8 mL of fresh pre-digestion buffer. Resume the program only after the buffer has been exchanged.

11.10 After the last washing step, remove the pre-digestion buffer and add 8 mL equilibration buffer. Resume the program only after the buffer has been exchanged.

12 **B) Enzymatic liver digest:**

12.1 Prepare the enzyme digestion mix for 1 perfusion during the equilibration phase.

A	B
Enzyme D	110 µL
Enzyme R	110 µL
Enzyme A	30 µL
Warm equilibration buffer	9 ml

12.2 After the equilibration step, remove the equilibration buffer and add 8 mL enzyme digestion mix. Resume program only after buffer has been exchanged.

12.3 After the enzymatic perfusion step, the program ends. Do not discard the used enzyme digestion mix.

13 **C) Mechanical dissociation of digested liver:**

13.1 Detach the heating unit and GentleMACS perfuser from the GentleMACS instrument.

13.2 Unscrew the lid and transfer the lid-clamp-grid assembly into a 60 mm tissue culture dish.

13.3 Pour the used enzyme digestion mix from the base into a GentleMACS C Tube and discard the base.

13.4 Push up the clamp using tweezers and transfer the tissue from the grid into the C Tube containing the used enzyme digestion mix. Discard the lid with attached clamp-grid assembly.

13.5 Tightly close the C Tube and attach it upside down onto a regular GentleMACS sleeve of the GentleMACS Dissociator.

Note

Exchange the Perfusion sleeves with the regular GentleMACS Sleeves

13.6 Run the GentleMACS program LIPK_HR_1.

Note

- Disruption of the perfused liver with manual methods might decrease the yield and viability of the isolated cells.
- If a significant amount (>30%) of undissociated liver is still attached to the rotor of the C Tube after the dissociation step, open the C Tube and transfer the undissociated liver tissue back into the liquid. Close the C Tube and run the GentleMACS Program for another 30 seconds.

13.7 Put a 15 mL tube On ice and place a MACS SmartStrainer (100 µm) on it.

Note

Important to work On ice from this step onwards!

13.8 After termination of the program, detach the C Tube from the GentleMACS Dissociator.

13.9 Open the tube and transfer the cell suspension onto the MACS SmartStrainer (100 µm).

13.10 Wash the C Tube with 6 mL of liver wash buffer and transfer this onto the MACS SmartStrainer (100 µm).

13.11 Discard the MACS SmartStrainer (100 µm) and close the 15 mL tube.

13.12 Gently invert the tube several times to ensure proper mixing of the liver wash buffer and the digested liver solution.

13.13 Keep the cells On ice until further processing.
