# Supplementary material for: Robust isolation protocol for mouse leukocytes from blood and liver resident cells for immunology research
Source: PLoS One. 2024 Aug 22;19(8):e0304063. doi: 10.1371/journal.pone.0304063 (PMC11340898; doi:10.1371/journal.pone.0304063)
Supplement: S3 File — (PDF) [file pone.0304063.s017.pdf]

Apr 12, 2024

Ex vivo cell isolation

In 1 collection

DOI
dx.doi.org/10.17504/protocols.io.q26g71b1kgwz/v1

Dorien De Pooter[1], Ben De Clerck[1], Koen Dockx[2], Domenica De Santis[2], Sarah Sauviller[1], Pascale Dehertogh[1], Matthias Beyens[3], Isabelle Bergiers[3], Isabel Nájera[4], Ellen Van Gulck[1], Nádia Conceição-Neto[1], Wim Pierson[1]

[1]ID Discovery, Infectious Diseases Therapeutic Area, Janssen Research and Development, Beerse, Belgium;
[2]Charles River Laboratories, Beerse, Belgium;
[3]Discovery Technologies & Molecular Pharmacology, Therapeutics Discovery, Janssen Research and Development, Beerse, Belgium;
[4]ID Discovery, Infectious Diseases Therapeutic Area, Janssen Research and Development, California, Brisbane, USA

Wim Pierson
ID Discovery, Infectious Diseases Therapeutic Area, Janssen ...

OPEN ACCESS

DOI: **dx.doi.org/10.17504/protocols.io.q26g71b1kgwz/v1**

Protocol Citation: Dorien De Pooter, Ben De Clerck, Koen Dockx, Domenica De Santis, Sarah Sauviller, Pascale Dehertogh, Matthias Beyens, Isabelle Bergiers, Isabel Nájera, Ellen Van Gulck, Nádia Conceição-Neto, Wim Pierson 2024. Ex vivo cell isolation. **protocols.io** **https://dx.doi.org/10.17504/protocols.io.q26g71b1kgwz/v1**

License: This is an open access protocol distributed under the terms of the **Creative Commons Attribution License**, which permits unrestricted use, distribution, and reproduction in any medium, provided the original author and source are credited

Protocol status: Working

Created: April 01, 2024

Last Modified: April 12, 2024

Protocol Integer ID: 98107

Abstract

This protocol details ex-vivo cell isolation.

Materials

Reagents:

- RPMI1640 medium with L-glutamine (Lonza, BE12-702F)
- FCS, frozen (0.22μm filtered Gibco by Thermo Fischer Scientific, 011-90005M)
- 10×PBS DPBS **Merck MilliporeSigma (Sigma-Aldrich) Catalog #**D1408
- Percoll **Merck MilliporeSigma (Sigma-Aldrich) Catalog #**17-0891-01
- 1× Dulbecco's PBS (without calcium magnesium) **Merck MilliporeSigma (Sigma-Aldrich) Catalog #**D8537
- Trypan Blue **Invitrogen - Thermo Fisher Catalog #**T10282
- 10% MACS BSA Stock Solution **Miltenyi Biotec Catalog #** 130-091-376
- William's E Medium, no phenol red **Thermo Fisher Catalog #**A1217601
- TheraPEAK ACK Lysing Buffer **Lonza Catalog #**BP10-548E

Reagent preparation:

1 PBS-2% FCS: Thaw an aliquot of filtered FCS and prepare a solution of 2% FCS (vol/vol) in 1x PBS.

2 33.75% Percoll gradient: Prepare an isotonic solution of 33.75% Percoll using 10x PBS and 1x PBS-2% FCS. Prepare this solution fresh and store at Room temperature and protect from light.

3 1x Debris removal solution: Prepare a 1:2 dilution of Debris removal solution in cold 1x PBS.

4 Trypan blue: Filter Trypan blue solution using a 100 µm cell strainer and dilute 1:2 in William's E medium.

5 MACS buffer: MACS buffer is a solution containing PBS pH 7.2, 0.5% BSA and 2 mM EDTA. Prepare this by diluting 10% BSA solution 1:20 and 0.5M EDTA 1:250 with 1x PBS.

6 PBS-0.04% BSA: PBS-0.04% BSA solution is prepared by diluting 10% BSA solution 1:250 in 1x PBS.

7 Culture medium: Add 50 mL of FCS to 500 mL of RPMI1640 with L-Glutamine.

Procedure: PART I

15m

8 **Liver: Separation hepatocytes from non-parenchymal cells**

8.1 Centrifugate the C-tubes containing liver dissociate at 50 x g, 4°C, 00:05:00 . Set the acceleration at 9 and brake at 5. 5m

8.2 After centrifugation, a big brown pellet should be visible which contains most hepatocytes. Take as much of the supernatant as possible without disturbing the hepatocytes (proceed to Section I.A). The supernatant contains the NPC; transfer it to a new 15 mL tube (proceed to Section I.B).

9 **I.A) Hepatocyte purification**

Note

CRITICAL! Always keep the hepatocytes On ice and work with cold buffers and solutions

9.1 Resuspend the hepatocyte pellet in 3 mL of William's E medium.

9.2 Underlay the cell suspension carefully with 6 mL of 1x debris removal solution by passively dispending it slowly with a pipette controller at the tube bottom to avoid mixing of the phases.

9.3 Centrifugate at 500 x g, 4°C, 00:10:00 10m

9.4 Aspirate the supernatant completely and resuspend the cell pellet to single cells in 1 mL cold William's E medium. After resuspension, add 4 mL cold William's E medium.

10 I.B) Non-Parenchymal Cells (NPC) purification

Note

IMPORTANT! For 10X sequencing, keep cells at Room temperature for the whole procedure.

10.1 Centrifugate the 15 mL tube containing the NPC at 400 x g, 00:05:00 , Room temperature . 5m

10.2 Discard supernatant by pouring, resuspend the NPC in 8 mL of 33.75% Percoll gradient.

10.3 Centrifugate at 700 x g, 00:12:00 , Room temperature . Put acceleration at maximum and brake at 3. 12m

10.4 During centrifugation, prewet Celltrics 50 µm strainer with 500 µL PBS-2% FCS.

10.5 Carefully remove the 15 mL tubes from the centrifuge to avoid sinking of the hepatocytes.

10.6 Remove the floating debris and hepatocytes carefully with a pipet without disturbing the pellet. Remove as much of the supernatant as possible.

> **Note**
>
> The type of downstream assay will determine the following steps.

11 **I.B.1) Flow Cytometry**

11.1 Resuspend the NPC in 1 mL PBS-2% FCS and transfer cells over 50 µm cell strainer in a new 5 mL polystyrene round bottom tube.

11.2 Rinse tube and filter with an additional 2 mL PBS-2% FCS.

11.3 Centrifugate the solution at 400 x g, 4°C, 00:05:00 . 5m

11.4 Discard supernatant and resuspend in 1 mL PBS-2% FCS and count cells.

12 **I.B.2) For 10X sequencing:**

12.1 Resuspend pellet in 1 mL of cold ACK buffer and incubate for 00:05:00 On ice . 5m

12.2 To stop the lysis, add 2 mL of cold PBS and transfer cells over 50 µm cell strainer in a new 5 mL polystyrene round bottom tube.

12.3 Rinse the filter and tube with 500 µL cold PBS-2% FCS.

12.4 Centrifugate at 400 x g, 4°C, 00:05:00 . 5m

12.5 Discard supernatant and resuspend cells in the appropriate volume culture media for cell counting.
