# Supplementary material for: Robust isolation protocol for mouse leukocytes from blood and liver resident cells for immunology research
Source: PLoS One. 2024 Aug 22;19(8):e0304063. doi: 10.1371/journal.pone.0304063 (PMC11340898; doi:10.1371/journal.pone.0304063)
Supplement: S4 File — (PDF) [file pone.0304063.s018.pdf]

Apr 12, 2024

Blood sampling, cell isolation, single-cell GEM-generation, globin mRNA blockers and sequencing library preparation protocol

In 1 collection

DOI
dx.doi.org/10.17504/protocols.io.kxygxyeydl8j/v1

Dorien De Pooter[1], Ben De Clerck[1], Koen Dockx[2], Domenica De Santis[2], Sarah Sauviller[1], Pascale Dehertogh[1], Matthias Beyens[3], Isabelle Bergiers[3], Isabel Nájera[4], Ellen Van Gulck[1], Nádia Conceição-Neto[1], Wim Pierson[1]

[1]ID Discovery, Infectious Diseases Therapeutic Area, Janssen Research and Development, Beerse, Belgium;
[2]Charles River Laboratories, Beerse, Belgium;
[3]Discovery Technologies & Molecular Pharmacology, Therapeutics Discovery, Janssen Research and Development, Beerse, Belgium;
[4]ID Discovery, Infectious Diseases Therapeutic Area, Janssen Research and Development, California, Brisbane, USA

Wim Pierson
ID Discovery, Infectious Diseases Therapeutic Area, Janssen ...

OPEN ACCESS

DOI: dx.doi.org/10.17504/protocols.io.kxygxyeydl8j/v1

Protocol Citation: Dorien De Pooter, Ben De Clerck, Koen Dockx, Domenica De Santis, Sarah Sauviller, Pascale Dehertogh, Matthias Beyens, Isabelle Bergiers, Isabel Nájera, Ellen Van Gulck, Nádia Conceição-Neto, Wim Pierson 2024. Blood sampling, cell isolation, single-cell GEM-generation, globin mRNA blockers and sequencing library preparation protocol. **protocols.io**
https://dx.doi.org/10.17504/protocols.io.kxygxyeydl8j/v1

License: This is an open access protocol distributed under the terms of the **Creative Commons Attribution License**, which permits unrestricted use, distribution, and reproduction in any medium, provided the original author and source are credited

Protocol status: Working

Created: April 01, 2024

Last Modified: April 12, 2024

Protocol Integer ID: 98108

Abstract

This protocol details blood sampling, cell isolation, single-cell GEM-generation, globin mRNA blockers and sequencing library preparation.

Materials

Reagents:

- UltraPure 0.5M EDTA, pH 8.0 **Thermo Fisher Scientific Catalog #**15575-038
- Ethanol ≥70% (v/v) TechniSolv® **VWR International Catalog #**83801.360
- 1× PBS

Dulbecco's Phosphate Buffered Saline (without calcium and magnesium) **Merck MilliporeSigma (Sigma-Aldrich) Catalog #**D8537-1L

- Cellaca AOPI Viastain **Nexcelom Catalog #**CS2-0106-25mL
- 10% MACS BSA Stock Solution **Miltenyi Biotec Catalog #** 130-091-376
- HISTOPAQUE 1119 **Merck MilliporeSigma (Sigma-Aldrich) Catalog #**11191-100ML
- TheraPEAK ACK Lysing Buffer **Lonza Catalog #**BP10-548E
- Anti-Ter-119 MicroBeads, mouse **Miltenyi Biotec Catalog #**130-049-901
- MACS buffer: MACS buffer is a solution containing PBS pH 7.2, 0.5% BSA and 2 mM EDTA. Prepare this by diluting 10% BSA solution 1:20 and 0.5M EDTA 1:250 with 1x PBS.
- PBS-0.04% BSA: PBS-0.04% BSA solution is prepared by diluting 10% BSA solution 1:250 in 1x PBS.
- FastSelect globin mRNA blockers 1X (QIAseq FastSelect – Globin Kit, Qiagen GmbH)
- Chromium Next GEM Single Cell 5' Reagent Kits v2 (Dual Index) (10x Genomics)
- Chromium Single Cell V(D)J Amplification Kits, Mouse (10x Genomics)
- Library Construction Kit (10x Genomics)
- Dual Index Kit TT set A, 96 rxns (10x Genomics)

Equipment:

- Mouse restrainer
- Electric fur clipper (Aesculap, GT415)
- Gauze pads
- Sharps container 1.5L (BD, 305624)
- Vortexer
- Centrifuge with swinging buckets
- Pipettors and tips
- Pipette controller
- Cellaca-MX-AOPI cell counter (Nexcelom)
- QuadroMACS Separator **Miltenyi Biotec Catalog #**130-090-976)
- Chromium Controller (10x Genomics)
- Chromium X (10x Genomics)
- 10x Vortex Adapter (10x Genomics)
- Chromium Next GEM Secondary Holder (10x Genomics)
- 10x Magnetic Separator (10x Genomics)
- Veriti 96-Well Thermal Cycler (Thermo Fisher Scientific)
- NovaSeq6000 platform (PE150) (Illumina)

Materials:

- Microlance 3 - 25G x 5/8" (0.5 x 16mm) needle (BD, 300600)
- Microvette capillary blood system, CB300 K2E, 0.3ML **Sarstedt Catalog #**16.444
- Microvette capillary blood system, CB300 Z, 0.3 mL **Sarstedt Catalog #**16.440
- Pluristrainer mini 70 µm filter (Puriselect, 43-10070-40)
- Celltrics 50 µm strainer (Sysmex, 04-004-2327)
- Falcon 50 mL Polypropylene conical Tube (Falcon, 352070)
- Falcon Round-Bottom polystyrene tubes 5 mL (Falcon, 352054)
- LD columns **Miltenyi Biotec Catalog #**130-042-901
- Cellaca counting plates (Nexcelom, CHM24-A100-004)
- Chromium Next GEM Chip K Single Cell (10x Genomics)

Blood sampling

1 Place the animal in the restrainer so that only one of the two the hind legs and tail are free. Stretch out the leg.

2 Remove the fur from the lateral side of the hind leg using the electric clipper.

3 Locate the lateral saphenous vein (If necessary: swab the skin with a small amount of ethanol to help visualize the vein).

4 Puncture the vein with the needle at a 90° angle at the most distal visible site.

5 Collect drops of blood until 200 µL is collected in the respective capillary tube.

Note

For whole blood: Microvette CB300 K2E; for serum: Microvette CB300 Z.

6 Use a dry piece of gauze to apply pressure to the puncture site until bleeding stops.

7 Remove the mouse from the restrainer.

8 Dispose the needle in an approved sharps container.

9 Close the trumpet-shaped side of the tube by pushing the cap.

- For leukocyte isolation: add extra 3 µL of EDTA solution to the tube, close the upper cap of the tube and vortex thoroughly.
- For serum analysis: close the upper cap of the tube. Do not vortex.

10 Store the tubes at Room temperature until further processing.

ACK lysis buffer selection

40m

11

Note

Important! Use all reagents at Room temperature .

Pipet the blood out of the capillary tubes and transfer the entire volume of blood, ~150 µL to a 50 mL tube.

12 Add 2 mL of ACK buffer (1x) and incubate the sample on a rocker for 00:05:00 at Room temperature . 5m

13 Add 10 mL of 1x PBS to the top of the tube to stop the lysis reaction and centrifuge the lysate sample at 400 x g, Room temperature, 00:05:00 . 5m

14 Pour off the supernatant. Add 10 mL PBS. Gently pipette 10 times to resuspend the pellet.

15 Filter the cell suspension through a 70 µm cell strainer and centrifuge at 400 x g, Room temperature, 00:05:00 . 5m

16 Pour off the supernatant and add 1 mL of ACK buffer to the pellet and incubate on a rocker for 00:05:00 Room temperature . 5m

17 Add 10 mL of 1x PBS to the top of the tube to stop the lysis reaction and centrifuge the lysate sample at 250 x g, Room temperature, 00:10:00 . 10m

18 Pour off the supernatant and perform a second wash with 10 mL of PBS and centrifuge at 250 x g, Room temperature, 00:10:00 . 10m

19 Pour off the supernatant and resuspend the pellet in equal volume of PBS-0.04% BSA to original whole blood volume.

20 Count cells using the Cellaca or Luna cell counter.

Anti-Ter119 Microbeads selection 45m

21

Note

Important! Use all reagents at Room temperature .

Transfer the blood from the capillary tubes over a 70 µm pluristrainer placed on a 5 mL round-bottom tube to remove any clots.

22 Rinse the original blood tubes twice with 100 µL of MACS buffer and rinse the filter with 3 mL MACS buffer.

23 Centrifugate the samples at 400 x g, Room temperature, 00:05:00 .

5m

24 Pipet off the supernatant (~ 3.3 mL) with a P1000 pipettor.

25 Briefly vortex the Anti-Ter119 microbeads and add 200 µL of beads. Mix well and incubate for 00:15:00 Room temperature .

15m

26 Add 3.5 mL of MACS buffer, mix the solution by inverting and centrifugate at 300 x g, Room temperature, 00:10:00 .

10m

27 Prepare the LD columns by putting them in the magnetic field of a QuadroMACS Separator and rinse columns with 2 mL MACS buffer; discard flowthrough and place a new 5 mL round-bottom tube under the columns.

28 From the centrifugated blood, remove supernatant using a P1000 pipettor and resuspend cells in 1000 µL of MACS buffer.

29 Apply the cell suspension onto the column and collect the unlabeled cells which pass through.

30 Wash tube that contained the blood-bead mixture with 1 mL of buffer and add to column. Add an additional 1 mL of MACS buffer to elute all the cells.

31 Mix the effluent with cells of interest, by inverting and spin down at 250 x g, Room temperature, 00:10:00 .

10m

32 Pour the supernatant and resuspend the cells in 4 mL of MACS buffer.

33 Spin down cells at 400 x g, Room temperature, 00:05:00 . Pour supernatant and resuspend pellet in cell culture media (500 µL) or 150 µL of PBS-0.04% BSA for a total volume of 200 µL . 5m

34 Count cells using the Cellaca or Luna cell counter.

ACK and Anti-Ter119 Microbeads selection

1h 5m

35

Note

Important! Use all reagents at Room temperature .

Pipet the blood out of the capillary tubes and transfer the entire volume of blood, ~150 µL to a 50 mL tube.

36 Add 2 mL of ACK buffer (1x) and incubate the sample on a rocker for 00:10:00 Room temperature . 10m

37 Add 4 mL of PBS to the top of the tube to stop the lysis reaction and centrifuge the sample at 400 x g, Room temperature, 00:05:00 . 5m

38 Pour off the supernatant and add 4 mL of MACS buffer to wash the cells, centrifuge at 250 x g, Room temperature, 00:10:00 . 10m

39 Resuspend the pellet in 540 µL of MACS buffer and add 50 µL of Anti-Ter119 MicroBeads.

40 Mix well and incubate for 00:15:00 Room temperature . 15m

41 After incubation, add 3.5 mL of MACS buffer and centrifuge at 300 x g, Room temperature, 00:10:00 . 10m

42 After centrifugation, pour off the supernatant and resuspend cells in 500 µL MACS buffer.

43 Prepare the LD columns by putting them in the magnetic field of a QuadroMACS Separator, place a 50 µm cell strainer onto the columns and prepare filter and column by rinsing with 2 mL of MACS buffer; discard flowthrough and place a new 5 mL tube under the columns.

44 From the centrifugated blood, take off supernatant using a P1000 pipettor and resuspend cells in 1000 µL of MACS buffer.

45 Apply the cell suspension onto the column and collect the unlabeled cells which pass through.

46 Wash tube with 1 mL of buffer and add to column. Add an additional 1 mL of MACS buffer to elute all the cells. Collect the total effluent which contains the white blood cells.

47 Mix the suspension by inverting and spin the total effluent down at 250 x g, Room temperature, 00:10:00 . 10m

48 Pour the supernatant and resuspend the cells in 4 mL of MACS buffer.

49 Spin down cells at 400 x g, Room temperature, 00:05:00 . Pour supernatant and resuspend pellet in cell media (500 µL) or 150 µL of PBS-0.04% BSA for a total volume of 200 µL . 5m

50 Count cells using the Cellaca or Luna cell counter.

Histopaque-1119 selection 45m

51 **Note**

Important! Use all reagents at Room temperature .

Prepare 5 mL tube with 3 mL Histopaque-1119 gradient fresh on the day of the experiment.

52 Pipet the blood out of the capillary tubes and slowly add it to the top of the gradient, and centrifuge the tube for 400 x g, 00:30:00 with low deceleration for 00:10:00 Room temperature . 40m

53 Remove, slowly, the white ring of cells at the top of the gradient.

54 Spin down cells at 400 x g, Room temperature, 00:05:00 . 5m

55 Resuspend cells in 200 µL of PBS-0.04% BSA.

56 Count cells using the Cellaca or Luna cell counter.

Library preparation and sequencing

57 Evaluate cell viability and cell counts with acridine orange/propidium iodine (AO/PI) onto the Luna FX7 cell counter (Logos Biosystem).

58 Adjust the volume of the cell suspension to 1000 cells/µL in PBS-0.04% BSA.

59 For each sample, load 20,000 cells (supplementary table 1) into the Chromium Controller (10X Genomics) and partition into single 10X barcoded droplet according to the Chromium single cell 3' Gel Bead Kit v2 manufacturer's instructions.

59.1 For a selection of samples that underwent anti-Ter 119 Microbeads isolation protocol, use a modified master mix recipe: 1 µL of 0.1X FastSelect globin mRNA blockers 1X (QIAseq FastSelect – Globin Kit, Qiagen GmbH) was introduced at the expense of 1 µL of water according to manufacturer's instructions.

59.2 For a selection of other samples, use a modified GEM-RT incubation on a Veriti 96- well thermal cycler (Thermofisher), as shown below:

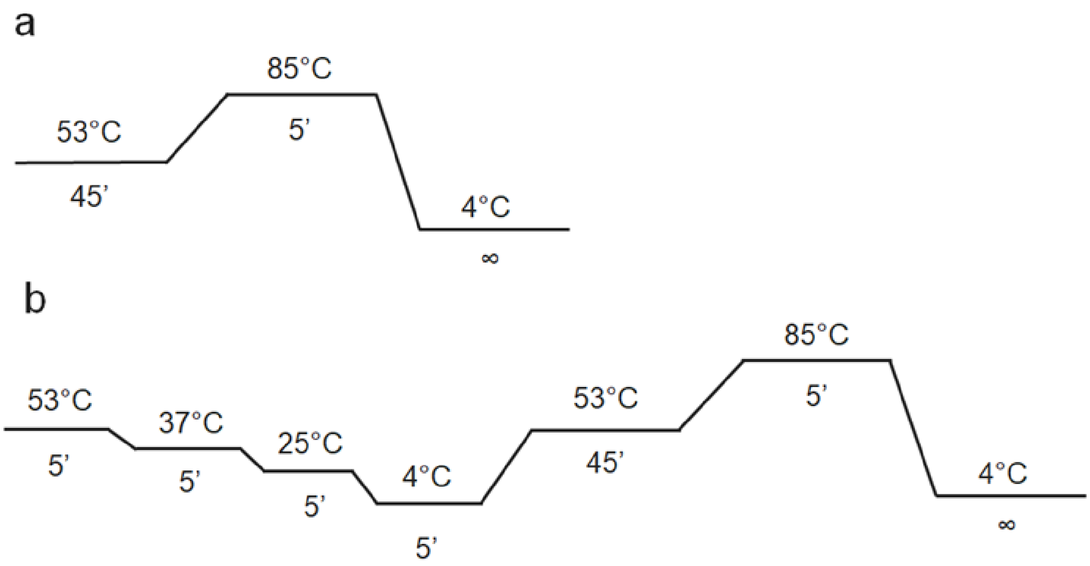

60 For the single-cell gene expression libraries, use the Chromium Single Cell 3' Reagent kit v2 (10X Genomics) according to manufacturer's instructions.

60.1 cDNAs recovered are amplified on Veriti 96-Well Thermal Cycler, the cDNAs products are amplified, cleaned up, quality controlled and quantified.

61 Add Illumina P5, P7, Read2 primers and Sample Index to the cDNAs products to generate sequencing libraries.

62 Perform quality control and quantify using a quantitative PCR (KAPA Biosystems Library Quantification Kit for Illumina platforms).

63 Load the sequencing libraries on a NextSeq2000 P3 flow cell with a sequencing depth of 50,000 reads per cell.
